# Genomic and Transcriptomic Resolution of Organic Matter Utilization Among Deep-Sea Bacteria in Guaymas Basin Hydrothermal Plumes

**DOI:** 10.3389/fmicb.2016.01125

**Published:** 2016-07-27

**Authors:** Meng Li, Sunit Jain, Gregory J. Dick

**Affiliations:** ^1^Institute for Advanced Study, Shenzhen UniversityShenzhen, China; ^2^Department of Earth and Environmental Sciences, University of MichiganAnn Arbor, MI, USA; ^3^Department of Ecology and Evolutionary Biology, University of MichiganAnn Arbor, MI, USA; ^4^Center of Computational Medicine and Bioinformatics, University of MichiganAnn Arbor, MI, USA

**Keywords:** metagenomics, metatranscriptomics, heterotrophic metabolism, bacteria, deep-sea hydrothermal plumes

## Abstract

Microbial chemosynthesis within deep-sea hydrothermal vent plumes is a regionally important source of organic carbon to the deep ocean. Although chemolithoautotrophs within hydrothermal plumes have attracted much attention, a gap remains in understanding the fate of organic carbon produced via chemosynthesis. In the present study, we conducted shotgun metagenomic and metatranscriptomic sequencing on samples from deep-sea hydrothermal vent plumes and surrounding background seawaters at Guaymas Basin (GB) in the Gulf of California. *De novo* assembly of metagenomic reads and binning by tetranucleotide signatures using emergent self-organizing maps (ESOM) revealed 66 partial and nearly complete bacterial genomes. These bacterial genomes belong to 10 different phyla: Actinobacteria, Bacteroidetes, Chloroflexi, Deferribacteres, Firmicutes, Gemmatimonadetes, Nitrospirae, Planctomycetes, Proteobacteria, Verrucomicrobia. Although several major transcriptionally active bacterial groups (Methylococcaceae, Methylomicrobium, SUP05, and SAR324) displayed methanotrophic and chemolithoautotrophic metabolisms, most other bacterial groups contain genes encoding extracellular peptidases and carbohydrate metabolizing enzymes with significantly higher transcripts in the plume than in background, indicating they are involved in degrading organic carbon derived from hydrothermal chemosynthesis. Among the most abundant and active heterotrophic bacteria in deep-sea hydrothermal plumes are Planctomycetes, which accounted for seven genomes with distinct functional and transcriptional activities. The Gemmatimonadetes and Verrucomicrobia also had abundant transcripts involved in organic carbon utilization. These results extend our knowledge of heterotrophic metabolism of bacterial communities in deep-sea hydrothermal plumes.

## Introduction

Deep-sea hydrothermal vents are typically distributed along the mid-ocean ridges throughout the world’s oceans, where hot and chemically reduced hydrothermal vent fluids mix with cold and oxidizing seawater, forming hydrothermal plumes that rise 100s of meters off the seafloor and disperse 100s of kilometers from their sources. The hydrothermal inputs, such as H_2_S, H_2_, CH_4_, NH_3_, Mn^2+^ and Fe^2+^, serve as energy sources that support microbial chemosynthesis (Winn et al., 1986; Deangelis et al., 1993). Evidence has suggested that this chemosynthesis is a significant source of organic carbon to the deep ocean (McCollom, 2000; Lam et al., 2004, 2008; Baker et al., 2012; Lesniewski et al., 2012). Although the global annual biomass production in hydrothermal plumes is a small fraction (~10^12^ g) of total primary production in the oceans, plume chemosynthesis may contribute a substantial fraction of organic carbon in the deep sea (McCollom, 2000). Therefore, understanding the cycling of organic carbon in deep-sea hydrothermal plumes is potentially important in terms of both deep-sea microbiology and microbial food web interactions.

Studies have begun to elucidate the importance and role of microorganisms and their metabolisms that operate within hydrothermal plumes (Lesniewski et al., 2012; Dick et al., 2013). Surveys of small subunit (SSU) ribosomal RNA (rRNA) genes using clone libraries (Sunamura et al., 2004; Dick and Tebo, 2010) and tag pyrosequencing (Sylvan et al., 2012) have revealed the composition of plume microbial communities. Metagenomic and metatranscriptomic results have provided insights into the roles of dominant microorganisms involved in oxidation of sulfur, hydrogen, methane, and ammonia in hydrothermal plumes, such as SUP05 (Anantharaman et al., 2013), *Methylococcaceae* (Lesniewski et al., 2012; Li et al., 2014a), Marine Group I Thaumarchaea (Baker et al., 2012) and SAR324 (Sheik et al., 2014). Studies also show that rare members of the plume microbial community such as *Alteromonadaceae* (Li et al., 2014b) and *Nitrospiraceae* (Baker et al., 2013) are potentially keystone species with roles in iron uptake and nitrite oxidation, respectively. These results have greatly enhanced our knowledge of deep-sea hydrothermal plume microbiology. However, previous studies focused on metabolisms related to autotrophy and inorganic electron donors, and little work has addressed the fate of organic carbon produced via chemosynthesis. Two recent studies presented metagenomic and metatranscriptomic evidence that widespread archaea (Li et al., 2015) and *Alteromonas* bacteria (Baker et al., 2013) play roles in scavenging a variety of organic compounds in the deep sea. Another recent study also inferred a microbial food web in which chemoautotrophy supports and heterotrophy in hydrothermal plumes at the Mid-Cayman Rise (Bennett et al., 2013). However, the broader role of bacteria in processing organic carbon in deep-sea hydrothermal plumes, both in terms of specific groups and pathways, remains unclear (Dick et al., 2013).

Guaymas Basin (GB), a submarine depression located on the seabed in the central area of the Gulf of California, hosts an unusual deep-sea hydrothermal system because of its location in a semi-enclosed basin and it proximity to the coast (Lonsdale and Becker, 1985). The ridge axis in GB has been blanketed by a 400 m layer of organic-rich sediment that chemically modifies hydrothermal fluids as they ascend toward the seafloor (Vondamm et al., 1985). The deep-sea vents inject hydrothermal solutions into the deep waters of a semi-enclosed basin, resulting in a plume where concentrations of methane (30 μM), ammonia (3 μM), and Mn (250 nM) are highly enriched over ambient deep sea levels (<0.5 μM, 0.25 μM, and 5 nM, respectively) at GB (Dick et al., 2009b). Methane and ammonia are energy sources that fuel substantial and diverse chemoautotrophy that provides a significant source of organic carbon to the deep oceans (Lam et al., 2004; Lesniewski et al., 2012). The objective of this study was to understand organic matter utilization by heterotrophic bacterial communities within GB hydrothermal plumes via shotgun metagenomic and metatranscriptomic sequencing. The results of this study shed light on the ecological and physiological properties of heterotrophic bacteria and highlight their critical role in oceanic carbon cycling.

## Materials and Methods

### Sample Collection, Extraction of Nucleic Acids, and DNA Sequencing

Samples from GB were collected on three cruises abroad the R/V New Horizon in 2004 and 2005 as described previously (Dick and Tebo, 2010). Cells for DNA and RNA extraction were filtered directly from the bottle onto a 142 mm 0.2 mM polycarbonate filter membrane by N_2_ gas pressure filtration at <5 psi. Filters were preserved immediately in RNA later (Ambion, Austin, TX, USA) as recommended by the manufacturer, incubated at 4°C overnight, then stored at -20°C for the duration of the cruise and at -80°C upon return to shore. To minimize the degradation of RNA during collection, samples were kept under *in situ* conditions (cold and dark) during CTD retrieval and then processed immediately on deck. Although, we cannot exclude the possibility that some changes in the RNA pool occurred during sample collection (it took approximately one hour to recover CTDs from 2000 m) and processing, these artifacts should affect all samples evenly and are circumvented to some extent by (i) the vast majority of recovery time is under water at temperatures that do not deviate substantially from those of plume waters, and (ii) the comparative nature of this study (plume versus background). Metadata and chemical/physical characteristics of samples are present in Supplementary Table S1 and more detail as previous reports (Lesniewski et al., 2012; Anantharaman et al., 2013).

DNA was extracted in June of 2012 as described in detail previously (Dick and Tebo, 2010; Lesniewski et al., 2012; Anantharaman et al., 2013; Li et al., 2015). Briefly, ¼ filters were cut into small pieces and cells were lysed with bead beating and a chemical solution (300 mM EDTA, 300 mM NaCl, 300 mM Tris, pH 7.5, 1.5% SDS, 50 mM DTT, 0.5 N acetate and lysozyme). DNA was then purified with Montage PCR purification filter units and eluted in TE buffer. RNA was extracted in June 2012 from 1/4 filters with the mirVana mRNA Isolation kit (Ambion), treated with DNase I to remove DNA, and concentrated and re-purified using the RNeasy MinElute Kit (Qiagen, Valencia, CA, USA). Total RNA was quantified by RiboGreen (Invitrogen, Carlsbad, CA, USA). RNA amplification by random priming and complementary DNA (cDNA) synthesis was performed as described previously (Frias-Lopez et al., 2008; Shi et al., 2009; Lesniewski et al., 2012). Shotgun sequencing of DNA and cDNA were performed with Illumina HiSeq2000 PE 100 with HiSeq Cluster Kit v4 (San Diego, CA, USA) at the University of Michigan Sequencing Core.

### *De Novo* Metagenomic Assembly and Gene Annotation

Metagenomic *de novo* assembly was performed as described previously (Li et al., 2015). In brief, short DNA reads were dereplicated (100% identity over 100% lengths) and trimmed using Sickle^1^. The trimmed and dereplicated pair-end short reads were used for *de novo* assembly by IDBA-UD with the following parameters: --mink 50, --maxk 92, --step 4 or 6, --min_contig 500. Gene calling and annotations were done using the DOE Joint Genome Institutes (JGI) Integrated Microbial Genomes pipeline^2^.

### Metagenome Binning and Identification of Bins

Assembled metagenomic sequences were assigned into putative taxonomic groups by binning with emergent self-organizing maps (ESOM) based on the analysis of sequence tetranucleotide frequencies (Dick et al., 2009a; cutoffs: minimum scaffold size = 5 Kb, maximum scaffold size = 10 Kb). To identify appropriate taxonomic levels of each genomic bins, we used following order of workflow: (i) identification of SSU rRNA genes on scaffolds by BLASTN (Altschul et al., 1990) against the Silva SSU Database version 115 (Quast et al., 2013); (ii) ESOM binning with reference genomes; (iii) taxonomic clustering of annotated genes by protein UBLAST with the IMG database (>50%) of identified open reading frames (Markowitz et al., 2008). Some genomic bins with multiple closely related genomes based on the presence of multiple copies of single-copy conserved genes were further separated by plotting differential coverage of scaffolds and their GC contents (Supplementary Table S2). The completeness and contamination of genomes within bins were estimated by counting single-copy conserved genes using CheckM with default setting, which is an automated method for assessing the quality of microbial genomes recovered from isolates, single cells, and metagenome (Parks et al., 2015; Supplementary Table S3).

### Identification of Key Functional Genes

For genes encoding peptidases and carbohydrate metabolizing enzymes, all annotated genes in bacterial genomes were searched against public databases of peptidases (MEROPS; Rawlings et al., 2014) and Carbohydrate-Active enZYmes (CAZYs; Lombard et al., 2014) with E-value < 10^-10^ by BLASTP. To further improve the accuracy after this screening, all hits were also compared to the non-redundant NCBI protein database (version 092012). Only those that had top hits to peptidases and carbohydrate metabolizing enzymes [glycoside hydrolases (GHs); polysaccharide lyases (PLs); carbohydrate esterases (CEs); auxiliary activities (AAs)] were considered. The extracellular peptidases were further confirmed based on the identification of extracellular transport signals using SignaIP (Petersen et al., 2011), POSRTb (Yu et al., 2010) or PRED-SIGNAL (Bagos et al., 2009), and these tools are widely used to predict the presence and location of signal peptides and their cleavage sites in amino acid sequences from different organisms. Genes related to carbohydrate metabolizing enzymes were further classified into different groups according the prediction of CAZYs (Lombard et al., 2014).

### Analysis of Transcript Abundance of Genomic Bins and Functional Genes

Abundance of transcripts for each genomic bins or functional genes was determined by mapping all non-rRNA transcripts to the assembled fragments or functional genes using Burrows–Wheeler Aligner (BWA) with default settings and normalizing to their sequence length and total number of non-rRNA transcripts in each metatranscriptomic library (Li and Durbin, 2009).

### Sequence Alignment and Phylogeny

Alignment of ribosomal protein S3, 16S rRNA genes were performed by MUSCLE using default parameters followed by manual refinement (Edgar, 2004). Phylogenetic analysis of representative ribosomal protein S3 were inferred by Maximum Likelihood implemented in Mega 6.0 (Tamura et al., 2013) using the Tamura-Nei and passion model after testing by ProTest (Darriba et al., 2011) and bootstrapped 1000 times. The phylogeny of 16S rRNA genes were inferred by maximum likelihood implemented in RaxML using the GAMMAGTR algorithm and bootstrap 1000 times in ARB software (Ludwig et al., 2004).

### Data Archiving

Scaffolds from metagenomic assembly are available from DOE JGI-IMG/MEG with Taxon Objects ID 3300001683 and NCBI with accession number LWDU00000000. The original short reads of metagenome and metatranscriptomes are available from NCBI with accession number SRP075597, SRX134768, and SRX134769, respectively.

## Results

### Genomic Reconstruction and Identification

**FIGURE 1:**
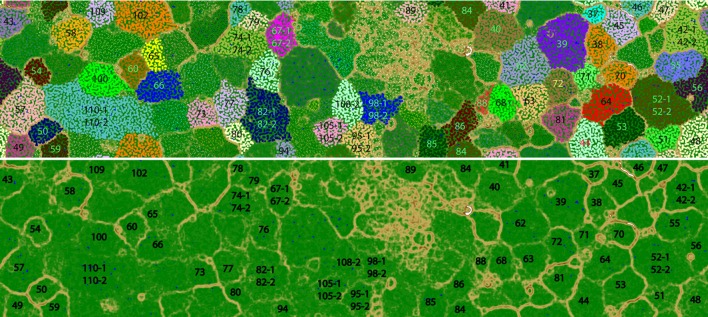
**Tetra-nucleotide ESOM binning map of Guaymas Basin plume metagenomic assembly. (Top)** Each point on the map represents a contig (>5 kb) or contig fragment generated *in silico* (5–10 kb). All identified bins are uniquely color coded as indicated. The ESOM map displayed is tiled and torroidal (that is, continuous from top to bottom and left to right). Note that in a few cases (Bin42, Bin52, Bin67, Bin74, Bin82, Bin95, Bin98, Bin105, Bin108, Bin110) multiple closely related bins fall within one large cluster on this map. These bins were delineated based on differential coverage plots. **(Bottom)** the same ESOM image, without data points shown, highlighting the topographic structure used to delineate bins. Higher “elevations” (brown, white) indicate large tetranucleotide frequency distances between data points, representing boundaries between genomic bins. Lower elevations (green, blue”) indicate small tetranucleotide frequency distances between data points, representing the continuity of tetranucleotide frequency space within genomes.

**FIGURE 2:**
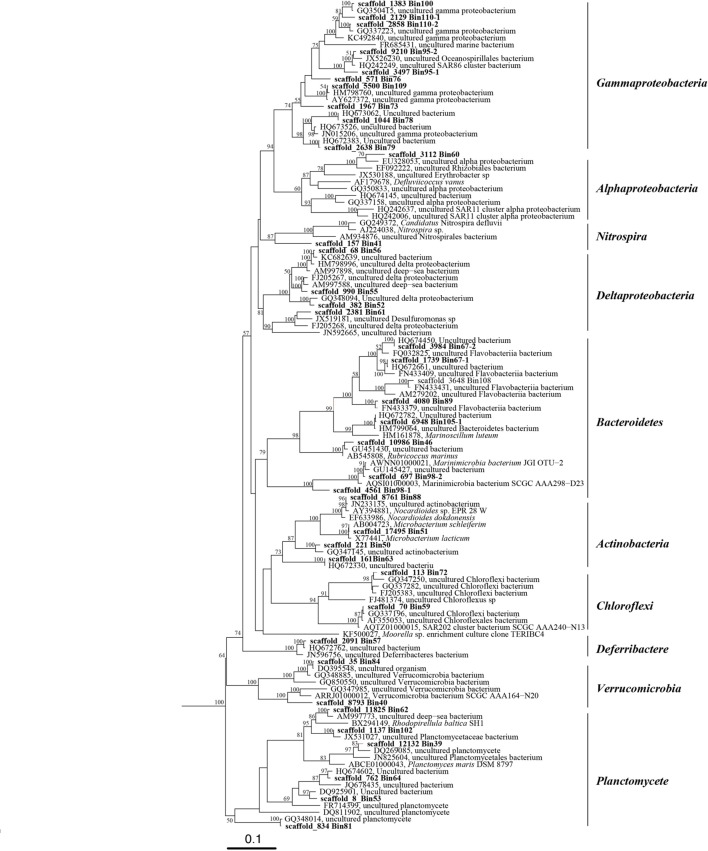
**Phylogenetic tree of 16S rRNA genes from bacterial genomic bins and their closely related sequences in public database.** This tree was generated using the maximum likelihood method in the ARB alignment (RAxML, ARB package) and phylogeny software package with 1000 time bootstraps; only bootstraps >50% are shown in the tree.

The number of DNA and cDNA short reads from plume and background are listed in Supplementary Table S1. Metagenomic assembly and binning by tetranucleotide frequencies coupled with coverage and GC content resulted in a total of 74 bacterial genomes (**Figure 1**; Supplementary Table S2). Among them, eight genomes predicted by the CheckM package to have relative high contamination were removed from further analysis. Of the remaining 66 genomes, 53 and 42 of these bacterial genomes were estimated to be >60 and >80% complete, respectively (Supplementary Table S3). To determine the taxonomic identities of these bacterial genomes, 16S rRNA genes (>500 bp) identified within the genomes (38 genomes) were used for phylogenetic analysis (**Figure 2**). For remaining genomes (28 genomes), we used phylogeny of ribosomal protein S3 (Supplementary Figure S1) and the binning of reference genomes to confirm their taxonomic identities (Castelle et al., 2013; Hug et al., 2013). These results indicated that the 66 genomes belong to 10 different bacterial phyla, including Actinobacteria (six genomes), Bacteroidetes (seven genomes), Chloroflexi (three genomes), Deferribacteres (three genomes), Firmicutes (one genome), Gemmatimonadetes (one genome), Nitrospirae (two genomes), Planctomycetes (seven genomes), Proteobacteria (*Alphaproteobacteria*, six genomes; *Betaproteobacteria*, one genome; *Deltaproteobacteria*, five genomes; *Gammaproteobacteria*, 20 genomes), and Verrucomicrobia (four genomes; Supplementary Table S2).

### Comparison of Genomic and Transcriptomic Abundance of Bacterial Members

**FIGURE 3:**
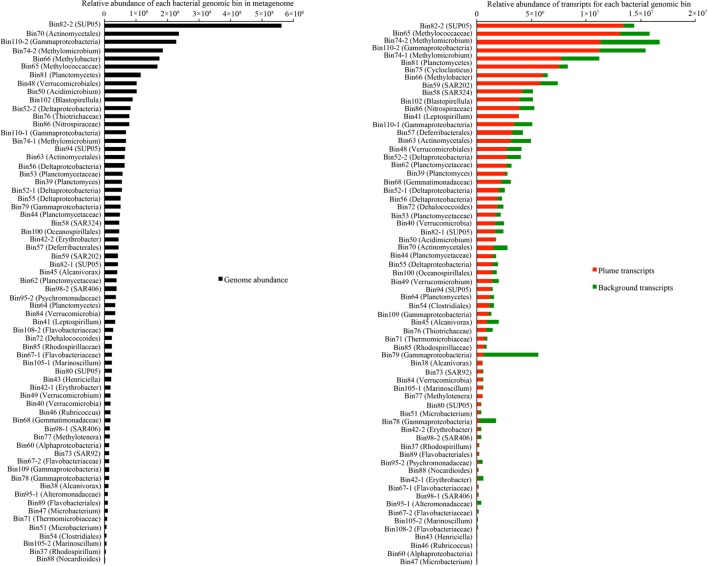
**Relative abundance of each bacterial group in metagenome and metatranscriptome in the GB plume and background.** Abundance is the number of DNA or cDNA reads mapped to mRNA genes in each genome, normalized to the length of the genes and total number of reads in metagenome and metatranscriptomes.

To compare the genomic and transcriptomic abundance of these genomic bins, DNA and cDNA reads were mapped to the assembled sequences and results were normalized to the sequence length and total number of DNA and cDNA short reads in metagenomic and metatranscriptomic libraries, respectively. From the genomic evidence, the most abundant bacteria in the GB plume included members of SUP05 (Bin82-2, Bin82-1, and Bin94), *Actinomycetales* (Bin70 and Bin63), *Gammaproteobacteria* methanotrophs (Bin75, Bin74-2, Bin74-1, Bin66, and Bin65), Planctomycetes (Bin81, Bin102, Bin53, Bin39, Bin44, Bin62, and Bin64), Verrucomicrobia (Bin48, Bin84, Bin49, and Bin40), *Acidimicrobium* (Bin50), and unknown *Gammaproteobacteria* (Bin110-2; **Figure 3**). However, transcriptomic evidence indicated that members of SUP05, methanotrophs, unknown *Gammaproteobacteria*, Planctomycetes, SAR202 (Bin59), and SAR324 (Bin58) are the most transcriptionally active bacteria groups (>4 × 10^6^), while members of Verrucomicrobia and *Acidimicrobium* and have relative low transcripts (1.6~2.7 × 10^6^) in the GB plume and background (**Figure 3**). Interestingly, most of these bacteria had higher transcript abundances in the plume than in the background with few exceptions from two unknown *Gammaproteobacteria* (Bin78 and Bin79) and *Alteromonadales* (Bin95-1 and Bin95-2; **Figure 3**).

### Widespread Occurrence of Extracellular Peptidases in GB Hydrothermal Plume Bacteria

**FIGURE 4:**
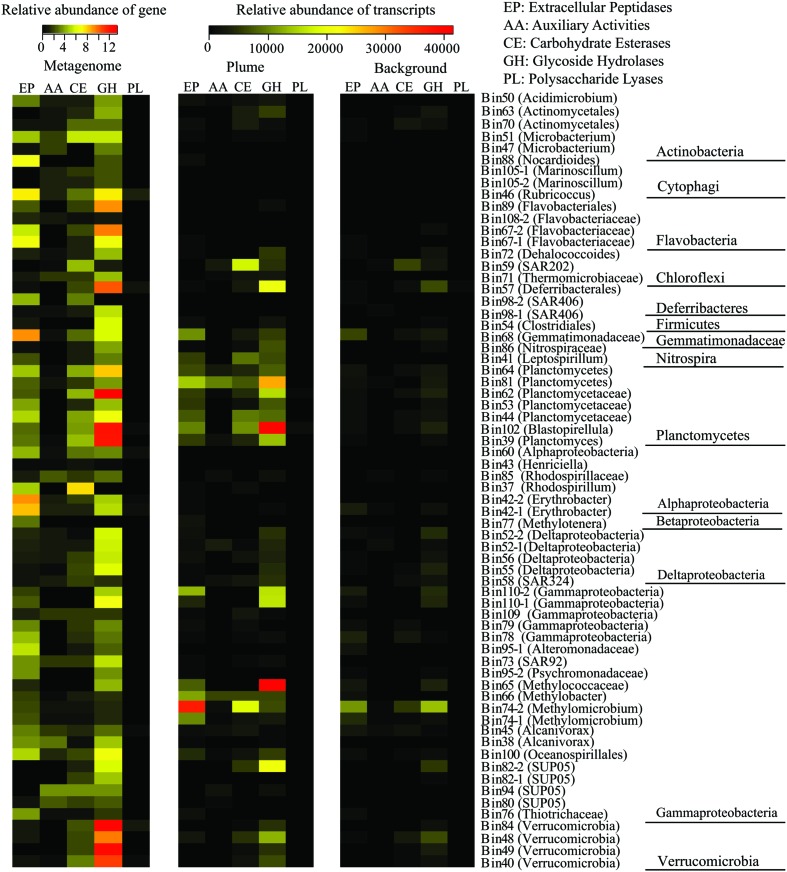
**Relative abundance of genes for extracellular peptidase (EP), auxiliary activities (AAs), carbohydrate esterases (CEs), glycoside hydrolases (GHs), and polysaccharide lyases (PLs) identified from each bacterial genome and their transcripts in the GB plume and background.** Gene abundance is the number of gene of each genomic bin that normalized to the size of the genomic bin, while transcript abundance is the number of cDNA reads mapped to the gene that normalized to the length of the gene and total number of reads in metatranscriptomes.

Of the 66 identified bacterial genomes, 59 genomes from 10 bacterial phyla contained multiple genes encoding extracellular peptidases, ranging from 0.3/Mb to 9.2/Mb. A member of Gemmatimonadetes (Bin68) had the most abundant extracellular peptidase genes (9.2/Mb), following by members of *Erythobacter* from *Alphaproteobacteria* (Bin42-2 and Bin42-1), *Rubricoccus* and *Flavobacteriaceae* from Bacteroidetes (Bin46, Bin67-1, and Bin67-2), and *Nocardioides* from Actinobacteria (Bin88; **Figure 4**; Supplementary Table S4). Transcripts for extracellular peptidases were also detected from 52 bacterial groups in the GB plume. Among them, members of *Methylococcaceae* (Bin74-2, Bin66, Bin74-1, and Bin65), Planctomycetes (Bin81, Bin102, Bin44, Bin64, and Bin39), Gemmatimonadetes (Bin68) and unknown *Gammproteobacteria* (Bin110-2) had the most abundant transcripts for extracellular peptidases in the GB plume, while members of *Methylococcaceae* (Bin74-2 and Bin74-1) and Gemmatimonadetes (Bin68) also have high extracellular peptidase transcript abundance in the background (**Figure 4**; Supplementary Table S4).

**FIGURE 5:**
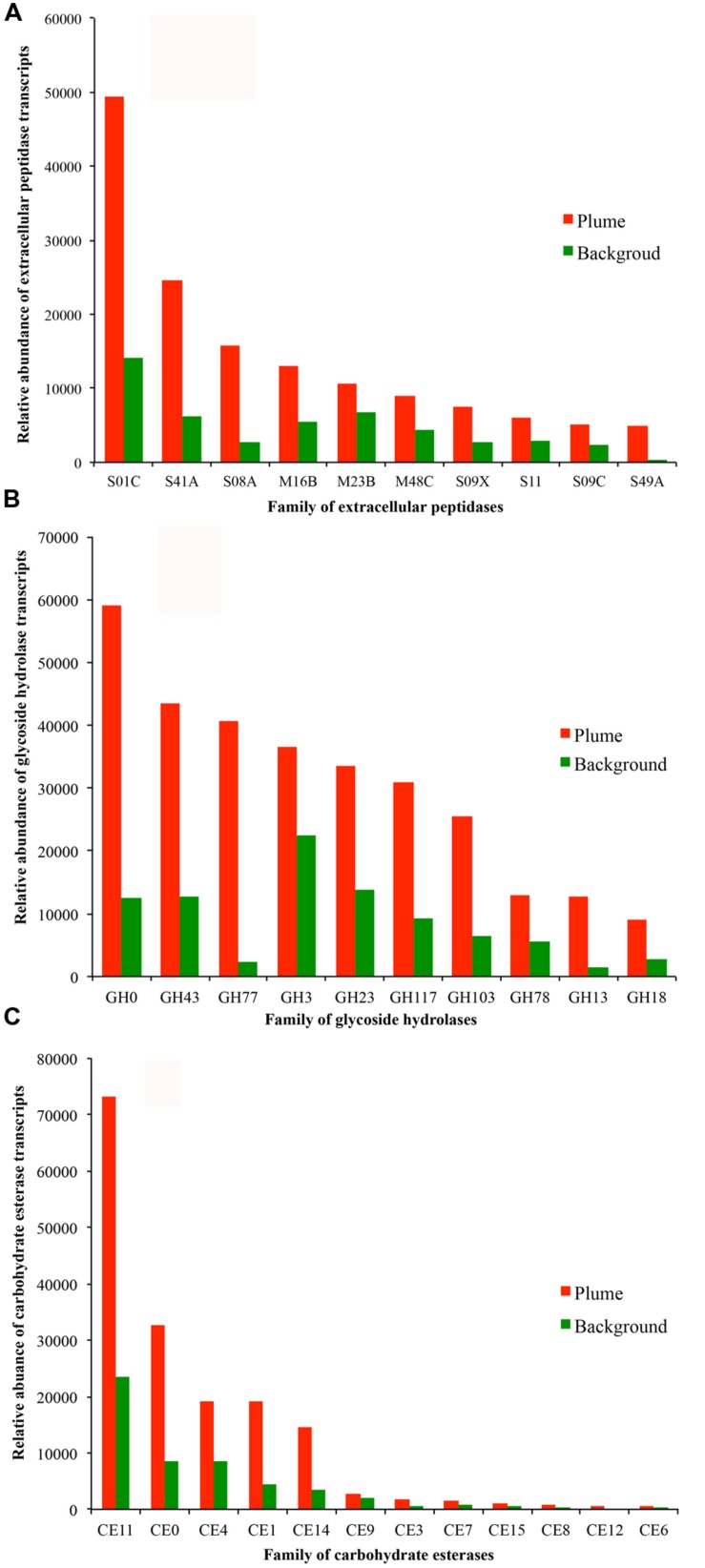
**Relative abundance of transcripts at the level of families of extracellular peptidases (A, top 10 families), GHs (B, top 10 families), and CEs (C) identified in the GB plume (red bar) and in the background (green bar).** More detailed information is provided in supplementary Figures S2.

The detected bacterial extracellular peptidases belong to 58 different families (**Figure 5A**). Among them, families S01C (*DegP* serine endopeptidases), S41A (C-terminal processing peptidase), S08A (subtilases), M16B (metalloendopeptidases), and M23B (lysostaphin) were the most abundant in the metatranscriptomes, representing 58.8 and 50.2% of all bacterial extracellular peptidase transcripts in the GB plume and background, respectively (**Figure 5A**). These five families of extracellular peptidases are related to serine endopeptidases (S01C, S41A, and S08A) and metalloendopeptidases (M16B and M23B), which cleave peptides at different sites of proteins, suggesting comprehensive protein degradation by deep-sea bacteria and potential use of protein as a growth substrate. Most extracellular peptidases had higher transcript abundance in the plume than in background, with only a few exceptions such as the families M16C (eupitrilysin), S49X (periplasmic serine protease), T02 (isoaspartyl aminopeptidases) and M03A (peptidyl-dipeptidase; **Figure 5A**).

### Ubiquitous Presence of Carbohydrate-Metabolizing Enzymes in GB Hydrothermal Plume Bacteria

In addition to extracellular peptidases, all bacterial genomes present in here were also searched for carbohydrate-metabolizing enzymes, including GHs, PLs, CEs, and AAs. A total of 65 bacterial genomes contain at least one copy of genes related to carbohydrate-metabolizing enzymes, suggesting the carbohydrate metabolisms are widespread in deep-sea bacteria identified in the GB hydrothermal plume (**Figure 4**; Supplementary Table S4). Members from Planctomycetes (Bin62, Bin102, Bin39, Bin64, and Bin44), Verrucomicrobia (Bin84, Bin49, Bin40, and Bin48), Bacteroidetes (Bin67-2, Bin89, Bin46, Bin108-2, and Bin67-1), Deferribacteres (Bin57) and Gemmatimonadetes (Bin68) have the most abundant genes for GHs (>6/Mb), while members of *Alphaproteobacteria* (Bin37), Actinobacteria (Bin51), Planctomycetes (Bin39, Bin62, Bin64), and Chloroflexi (Bin59) have high CEs coding gene abundance (>4/Mb). For PLs and AAs, all bacterial genomes identified here had relatively low gene abundance (**Figure 4**). Transcripts for GHs and CEs were detected from 56 and 44 bacteria groups (**Figures 5B,C**), while only 5 and 31 bacteria groups have transcripts related to PLs and AAs in the GB hydrothermal plume (Supplementary Figure S2). Among these bacteria, members of *Methylococcaceae* (Bin65), Planctomycetes (Bin102, Bin81), SUP05 (Bin82-2), Deferribacteres (Bin57) and Verrucomicrobia (Bin48) have the most abundant transcripts for GHs, while members of *Methylococcaceae* (Bin74-2), SAR202 (Bin59), and Planctomycetes (Bin102 and Bin44) have high CE transcripts. Furthermore, GHs and CEs have much higher transcript abundance than those related to PLs and AAs (**Figure 4**; Supplementary Table S4).

Further analysis demonstrates that families of GH0, GH43, GH77, GH3, GH23, GH117, and GH103 for GH are the most abundant in the metatranscriptomes, representing 71.8 and 70.2% of all bacterial GH transcripts in the GB plume and background (**Figure 5B**). These dominant families are related to aldose 1-epimerases, arylsulfatases, sulfatases, beta-lactamases, beta-*N*-acetylglucosaminidases, lytic transglycosylases, *N*-acetyl-beta-hexosaminidases, and many uncharacterized GHs. While for CEs, families of CE11 and CE0 are the most abundant in the metatranscriptomes, responding to 63.0 and 60.5% of all bacterial CE transcripts in the GB plume and in the background (**Figure 5C**). Finally, similar with the extracellular peptidases, all detected families of GHs and CEs have much higher transcripts in the plume than in the background (**Figure 5**).

### Organic Matter Utilization of Planctomycetes, Gemmatimonadetes, and Verrucomicrobia in Deep-Sea Hydrothermal Plumes

**FIGURE 6:**
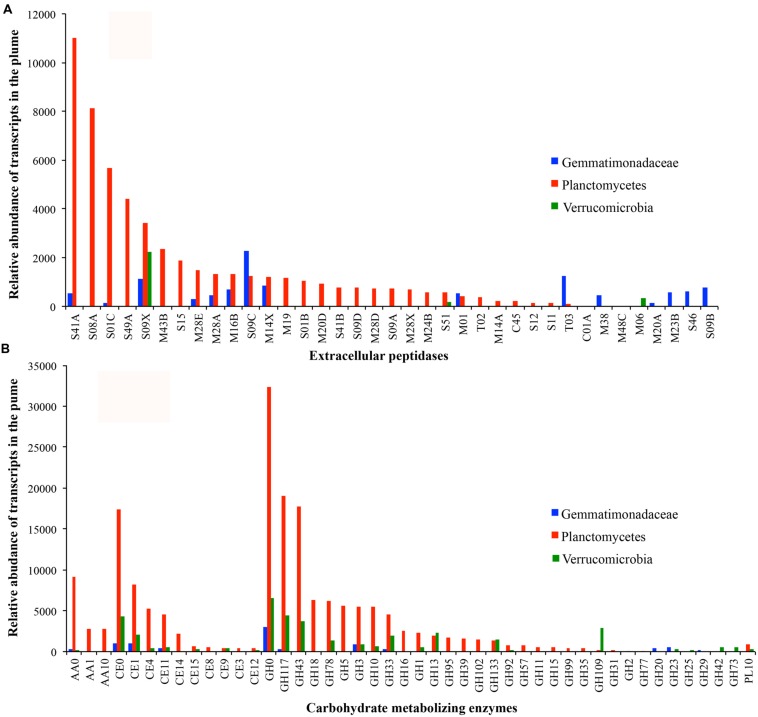
**Transcript abundance of extracellular peptidases (A) and carbohydrate metabolizing enzymes (B) in bacteria of Gemmatimonadetes (blue), Plantomycetes (red), and Verrucomicrobia (green) in the GB plume**.

To better understand the most active heterotrophic bacteria, the extracellular peptidases and carbohydrate-metabolizing enzymes in Planctomycetes, Gemmatimonadetes, and Verrucomicrobia were characterized further. Members of Planctomycetes are the most abundant and transcriptionally active heterotrophic bacteria in the GB hydrothermal plume (Supplementary Figure S3). Seven near-complete genomes of Planctomycetes with distinct genomic and transcriptomic abundance were identified (**Figure 6**). These seven genomes contained a total of 122 extracellular peptidase genes from 33 different families. Among them, transcripts for 27 families were detected, representing 27.9% of all bacterial extracellular peptidase transcripts in the GB plume (**Figure 6A**). For carbohydrate metabolizing enzymes, a total of 369 genes for 29 different families of GHs and 134 of CE genes belonging to 10 families were also identified in the seven Planctomycete genomes, representing 31.9 and 26.6% of all bacterial transcripts for GHs and CEs in the GB plume, respectively (**Figure 6B**). Similarly, transcripts involved in PLs and AAs identified in members of Planctomycetes also accounted for 35.4 and 69.3% of all related bacterial transcripts in the GB plume. These results indicate that members of Planctomycetes are involved in scavenging various organic matter in the GB plume. Interestingly, these Planctomycetes bacteria also have many genes and transcripts involved in the hydrolysis of sulfate esters, such as arylsulfatase, choline sulfatases and sulfatases, indicating Planctomycetes also respond for the utilization of organic sulfur compounds in the deep-sea (Supplementary Figure S4).

Only one genome related to Gemmatimonadetes was identified in the GB hydrothermal plume. This Gemmatimonadetes genome was estimated to be 85.8% complete and contained high abundance of genes for extracellular peptidases (the most of all bacterial genomes recovered) and carbohydrate metabolizing enzymes (**Figure 4**). Furthermore, this deep-sea Gemmatimonadetes bacterium also had high transcript abundance for carbohydrate metabolizing enzymes and extracellular peptidases, accounting for 1.5 and 5.5% of all bacterial carbohydrate metabolizing enzyme and extracellular peptidase transcripts, respectively (**Figure 6A**). For members of Verrucomicrobia, four genomes were identified in the GB hydrothermal plume, ranging in completeness from 44.4 to 99.8%. In these Verrucomicrobia, only eight extracellular peptidase genes were identified, but 59 genes encoding carbohydrate metabolizing enzymes were found, the most of all bacteria identified in this study. In addition, Verrucomicrobia bacteria had high abundance of transcripts for carbohydrate metabolizing enzymes, but relative lower transcripts for extracellular peptidases (**Figures 4** and **6**).

## Discussion

In the GB hydrothermal plume, the dominant active methanotrophs and chemolithotrophs produce labile organic matter that may support heterotrophic microorganisms (Baker et al., 2012; Lesniewski et al., 2012; Anantharaman et al., 2013; Li et al., 2014b). A recent study has reported the heterotrophic metabolisms of deep-sea archaea, indicating the critical roles of ubiquitous archaea in scavenging various organic matter (Li et al., 2015). To understand the bacterial heterotrophic metabolisms, we have reconstructed a total of 66 bacterial genomes from the GB hydrothermal plume through the combined use of shotgun metagenomic sequencing, *de novo* genomic assembly, tetranucleotide signature ESOM binning, and manual curation (**Figure 1**; Supplementary Table S2). The identified 66 bacterial genomes affiliate to 10 different bacterial phyla, indicating a diverse community structure and greatly expanding the genomic coverage of GB plume microorganisms. Furthermore, most of reconstructed bacterial genomes are nearly complete, including many uncharacterized deep-sea bacteria, such as members of Deferribacteres (Bin98-1, Bin98-2, and Bin57) and unknown *Deltaproteobacteria* (Bin52-1, Bin52-2, Bin55, Bin56, and Bin61; Supplementary Table S3). Thus, the data presented here provide substantial genomic and transcriptomic information to understand the function of bacteria in deep-sea hydrothermal plumes. We note that the metatranscriptomic data presented in this paper are in terms of *relative* abundance, thus we are unable to make inferences regarding levels of gene *expression*. Rather our results are in terms of relative abundance of transcripts, which provides a valuable measure of the relative contributions of genes and organisms to the pool of community transcripts.

Although the microbial community structures in the GB hydrothermal plume are similar to these in surrounding background waters (Dick and Tebo, 2010; Lesniewski et al., 2012), most of the bacteria in the plume had higher relative abundance of transcripts than in background, suggesting bacteria in the GB plume are stimulated by abundant substrates (**Figure 3**). Consistent with previous results, both genomic and transcriptomic evidence show that the most abundant and transcriptionally active bacterial groups were members of SUP05, methanotrophs, and SAR324, which display evidence of chemolithoautotrophic metabolisms (Baker et al., 2012; Lesniewski et al., 2012; Anantharaman et al., 2013; Li et al., 2014a; Sheik et al., 2014). However, the presence of genes encoding extracellular peptidases and carbohydrate metabolizing enzymes in most of bacterial genomes in the GB plume suggests that the potential for organic matter utilization in the deep-sea bacteria is also widespread (**Figure 4**). Taken together with the results of archaea in GB (Li et al., 2015), the utilization of organic matter is one of most common microbial metabolisms in the GB hydrothermal plume. Furthermore, transcriptomic evidence indicated that 66 bacterial groups from 10 bacterial phyla contain transcripts for different families of extracellular peptidases and carbohydrate metabolizing enzymes, suggesting these bacteria from different phyla are transcriptionally active for different organic matter utilization in the GB hydrothermal plume. Given the predominant chemolithoautotrophic metabolisms in the GB hydrothermal plume, the much higher abundance of transcripts of extracellular peptidases and carbohydrate metabolizing enzymes in the plume than in the background support that these bacteria may be degrading organic carbon derived from hydrothermal chemosynthesis.

The Planctomycetes are a bacterial phylum that is ubiquitous in natural environments. All currently described pure cultures of Planctomycetes are aerobic heterotrophs (the members of *Brocadiaceae* that conduct anaerobic ammonia oxidation are still not purified) capable of growth on several sugars and sugar alcohols, such as glucose, fructose, mannitol, xylose, ribose, and fucose (Youssef and Elshahed, 2014). Many grow exceptionally well on specific substrates, such as *N*-acetylglucosamine (NAG) presented as a side chain on mucin and chondroitin sulfate in nature. Furthermore, several described Planctomycetes species can metabolize polysaccharides, including starch, gelatin, and carboxymethyl cellulose (Schlesner et al., 2004; Kulichevskaya et al., 2009; Bondoso et al., 2011; Kulichevskaya et al., 2012). This preference for monomers and sulfated polymers has lead to suggestions that degradation of sulfated polymeric carbon, e.g., marine snow, is a natural role for Planctomycetes in various habitats (Glöckner et al., 2003; Woebken et al., 2007). Consistent with previous results, seven genomes of Planctomycetes identified in the GB hydrothermal plume contain abundant genes and transcripts for extracellular peptidases and carbohydrate metabolizing enzymes, particularly genes related to sulfatases, suggesting they perform important roles in organic carbon cycling in the GB plume (**Figure 6**).

Bacteria belonging to phylum Gemmatimonadetes have been identified as one of the top nine phyla found in soils, comprising ~2% of soil bacterial communities (Bossio et al., 1998; DeBruyn et al., 2011). Despite their frequency and persistent abundance in soils, information about this phylum in marine environments is quite limited. Currently, only few representatives from this phylum have been isolated and partially characterized (Joseph et al., 2003; Zhang et al., 2003; Davis et al., 2005). Members of Gemmatimonadetes are aerobic heterotrophs capable of utilization of multiple substrates, including east extract, polypepton, succinate, acetate, gelatin, and benzoate (Zhang et al., 2003). A recent study reported five genomes of Gemmatimonadetes from estuary sediments and found that members of Gemmatimonadetes contain a large number of genes of carbohydrate hydrolysis and protein degradation (Baker et al., 2015). Here, we have reconstructed a nearly complete (85.8%) genome of Gemmatimonadetes from deep oceans, which has the most abundant extracellular peptidase genes (9.2/Mb). Together with results from transcriptomes, as well as carbohydrate metabolizing enzymes, the data presented here strongly support Gemmatimonadetes bacteria as key mediators of the organic carbon cycle in the GB hydrothermal plumes (**Figure 6**).

The third interesting bacteria group are members belonging to the phylum of Verrucomicrobia, which are widely detected in different freshwater and marine habits (Wagner and Horn, 2006). Previous studies have confirmed that members of Verrucomicrobia are capable of heterotrophic, carbohydrate-degrading metabolisms (Arnosti, 2011; Steen et al., 2012; Cardman et al., 2014). The genomic evidence from a metagenome in Baltic seawater indicated that the genome of Verrucomicrobia bacteria encodes a diversity of GHs that likely allow degradation of various complex carbohydrates, such as cellulose, mannan, xylan, chitin, and starch (Herlemann et al., 2013). A recent report also proposed Verrucomicrobia are involved in polysaccharide hydrolysis in the water column and sediment (Cardman et al., 2014). Here, the four genomes of Verrucomicrobia contain diverse GH and CE genes, which also have relative high transcripts in the GB hydrothermal plume (**Figure 6B**), implying important roles of these microorganisms in the deep-sea carbon cycle.

## Conclusion

In summary, a total of 66 bacterial genomes from 10 different phyla in the GB hydrothermal plume were reconstructed through a combination with high-throughput sequencing, *de novo* genomic assembly and tetranucleotide binning. The widespread presence of extracellular peptidase and carbohydrate metabolizing enzyme genes in most of bacterial genomes suggests that the utilization of organic carbon is a common microbial metabolism in the GB hydrothermal plume. Furthermore, the higher relative abundance of transcripts of extracellular peptidases and carbohydrate metabolizing enzymes in the plume than in background indicate these bacteria might be involved in degradation of organic carbon derived from hydrothermal chemosynthesis. Hence, primary and secondary production in hydrothermal plumes may be tightly coupled. Although the mechanism of trophic transfer is unknown, the presence of abundant viruses that infect the autotrophs in GB and other hydrothermal plumes (Anantharaman et al., 2014) suggests that viral lysis is one potential source of carbon for heterotrophs. Among the most abundant and active heterotrophic bacteria in deep-sea hydrothermal plumes are members of the Planctomycetes, Gemmatimonadetes, and Verrucomicrobia. These results extend our knowledge of heterotrophic metabolism of bacterial communities within deep-sea hydrothermal plumes, highlighting their critical role in organic carbon cycling in the deep-sea hydrothermal plumes.

## Author Contributions

ML and GD designed the study; ML and SJ performed the genomic analyses. All authors wrote the manuscript and the Supplementary Materials.

## Conflict of Interest Statement

The authors declare that the research was conducted in the absence of any commercial or financial relationships that could be construed as a potential conflict of interest.

## Footnotes

1 https://github.com/najoshi/sickle

2 http://img.jgi.doe.gov/cgi-bin/w/main.cgi
