# Supplementary material for: Genomic and Transcriptomic Resolution of Organic Matter Utilization Among Deep-Sea Bacteria in Guaymas Basin Hydrothermal Plumes
Source: Front Microbiol. 2016 Jul 27;7:1125. doi: 10.3389/fmicb.2016.01125 (PMC4962555; doi:10.3389/fmicb.2016.01125)
Supplement: Supplementary file 1 [file Data_Sheet_1.PDF]

Table S1. Sample information of this study.

**Sample ID**	**Date**	**Sample type**	**Latitude/ Longitude**	**Depth (m)**	**tMn (nM)**	**dMn (nM)**	**Tem. (°C)**	**O2 (μM)**	**No. of sequence reads**[1]	**DNA or cDNA**
GOC11-1#2	11/07/2004	Neutrally buoyant plume	N 27°30.95 W 111°25.5	1,993	266	78	2.91	29.13	171,620,910 (5,715)	DNA
GOC12-27a#1	11/07/2004	Neutrally buoyant plume	N 27°30.360 W 111°20.818	1,950	288	40	2.93	27.70	206,157,516	cDNA
GOC12-8#12	11/07/2004	Above plume background	N 27°29.174 W 111°21.844	1,600	55	5	2.97	28.50	244,519,176	cDNA

Table S2. Details of identified bacterial genomic bins in the GB metagenome

Bin	Taxonomy	No. of Contigs	No. of Genes	No. of CDS	Avg. GC%	Total Length of Bin	Avg. Coverage
Bin37	Alphaproteobacteria; Rhodospirillales; Rhodospirillaceae; Rhodospirillum	97	813	793	61.11	642278	8.94
Bin38	Gammaproteobacteria; Oceanospirillales; Alcanivoracaceae; Alcanivorax	219	2273	2202	58.82	1982521	8.96
Bin39	Planctomycetes; Planctomycetia; Planctomycetales; Planctomycetaceae; Planctomyces	490	7552	6871	55.93	7646261	11.85
Bin40	Verrucomicrobia; Verrucomicrobiae; Verrucomicrobiales; Verrucomicrobia subdivision 3	409	3624	3421	48.98	3521385	8.46
Bin41	Nitrospirae; Nitrospira; Nitrospirales; Nitrospiraceae; Leptospirillum	46	2326	2232	49.25	2237706	24.30
Bin42-1	Alphaproteobacteria; Sphingomonadales; Erythrobacteraceae; Erythrobacter	200	2281	2806	64.54	2539479	11.43
Bin42-2	Alphaproteobacteria; Sphingomonadales; Erythrobacteraceae; Erythrobacter	36	3235	3120	64.82	3180590	23.35
Bin43	Alphaproteobacteria;Rhodobacterales; Hyphomonadaceae;Henriciella	190	3540	3438	60.07	3056955	10.01
Bin44	Planctomycetes; Planctomycetia; Planctomycetales; Planctomycetaceae	63	3315	3214	60.77	3896962	25.63
Bin45	Gammaproteobacteria; Oceanospirillales; Alcanivoracaceae; Alcanivorax	63	3536	3426	64.37	3512607	18.43
Bin46	Bacteroidetes; Cytophagia; Rhodothermaceae; Rubricoccus	250	2889	2808	71.02	2729017	10.26
Bin47	Actinobacteria; Actinobacteridae; Actinomycetales;Micrococcineae; Microbacteriaceae; Microbacterium	170	1933	1856	66.94	1605405	10.06
Bin48	Verrucomicrobia; Verrucomicrobiae; Verrucomicrobiales	369	3456	3359	59.45	3457916	51.20
Bin49	Verrucomicrobia; Verrucomicrobiae; Verrucomicrobiales;Verrucomicrobiaceae; Verrucomicrobium	203	2019	1981	57.96	1976049	17.15
Bin50	Actinobacteria; Actinobacteria; Acidimicrobiales; Acidimicrobiaceae; Acidimicrobium	42	1992	1920	55.97	1897076	87.11
Bin51	Actinobacteria; Actinobacteria; Micrococcales; Microbacteriaceae; Microbacterium	153	1260	1220	63.23	1089698	9.44
Bin52-1	Deltaproteobacteria	199	3859	3719	61.15	4045268	22.85
Bin52-2	Deltaproteobacteria	129	3649	3507	61.57	3835224	38.52
Bin53	Planctomycetes; Planctomycetia; Planctomycetales; Planctomycetaceae	17	3053	2953	64.18	3668126	31.88
Bin54	Firmicutes; Clostridia; Clostridiales	145	1364	1315	51.53	1179398	8.28
Bin55	Deltaproteobacteria	63	4642	4379	59.26	4702148	18.27
Bin56	Deltaproteobacteria	102	5327	5008	59.20	5043058	19.91
Bin57	Deferribacteres; Deferribacteres; Deferribacterales	393	6640	6242	56.24	6069167	10.72
Bin58	Deltaproteobacteria; SAR324 clade	168	3587	3429	56.39	3354330	20.88
Bin59	Chloroflexi; SAR202 clade	73	3293	3093	54.16	3017461	20.23
Bin60	Alphaproteobacteria	130	2705	2637	47.89	2428917	9.75
Bin62	Planctomycetes; Planctomycetacia; Planctomycetales; Planctomycetaceae	369	5700	5412	54.92	6129926	11.51
Bin63	Actinobacteria; Actinobacteria; Actinomycetales	74	3210	3048	55.13	3030256	27.38
Bin64	Planctomycetes; OM190	69	3302	3207	63.60	3966519	18.50

Bin65	Gammaproteobacteria; Methylococcales; Methylococcaceae	89	899	850	43.00	716645	317.58
Bin66	Gammaproteobacteria; Methylococcales; Methylococcaceae; Methylobacter	126	4057	3508	41.12	3355720	48.30
Bin67-1	Bacteroidetes; Flavobacteria; Flavobacteriales; Flavobacteriaceae	71	1631	1583	32.90	1626761	24.79
Bin67-2	Bacteroidetes; Flavobacteria; Flavobacteriales; Flavobacteriaceae	127	1502	1463	33.82	1476649	15.99
Bin68	Gemmatimonadetes; Gemmatimonadetes; Gemmatimonadales;Gemmatimonadaceae	72	2328	2255	52.93	2502548	12.84
Bin70	Actinobacteria; Actinobacteria; Actinomycetales ;Mycobacteriaceae	115	2010	1964	68.51	1812602	198.87
Bin71	Chloroflexi; Thermomicrobia; Thermomicrobiales; Thermomicrobiaceae	127	1425	1303	58.40	1165248	9.98
Bin72	Chloroflexi; Dehalococcoidia; Dehalococcoidales;Dehalococcoidaceae; Dehalococcoides	47	2783	2607	53.50	2551913	13.73
Bin73	Gammaproteobacteria; Alteromonadales; Alteromonadaceae; SAR92 clade	84	2368	2299	46.19	2290779	10.35
Bin74-1	Gammaproteobacteria; Methylococcales; Methylococcaceae; Methylomicrobium	151	2789	2530	40.34	2265828	31.63
Bin74-2	Gammaproteobacteria; Methylococcales; Methylococcaceae; Methylomicrobium	90	1629	1564	42.35	1426501	192.00
Bin76	Gammaproteobacteria;Thiotrichales;Thiotrichaceae	93	2926	2740	38.09	2458258	39.96
Bin77	Betaproteobacteria; Methylophilales; Methylophilaceae; Methylotenera	46	428	411	43.38	339439	61.22
Bin78	Gammaproteobacteria	70	1461	1413	41.31	1363631	15.58
Bin79	Gammaproteobacteria	47	1330	1287	37.16	1231487	63.77
Bin80	Gammaproteobacteria; Oceanospirillales; SUP05 cluster	63	1799	1740	42.94	1625629	15.36
Bin81	Planctomycetes; Pla3 lineage	155	2663	2547	57.39	2991918	71.79
Bin82-1	Gammaproteobacteria; Oceanospirillales; SUP05 cluster	101	1847	1793	37.80	1556371	27.98
Bin82-2	Gammaproteobacteria; Oceanospirillales; SUP05 cluster	84	1034	1011	39.01	833879	819.56
Bin84	Verrucomicrobia	44	3012	2923	44.61	3700935	18.76
Bin85	Alphaproteobacteria; Rhodospirillales; Rhodospirillaceae	127	3155	2996	41.22	2903704	13.12
Bin86	Nitrospirae;Nitrospira;Nitrospirales;Nitrospiraceae	225	2665	2459	42.23	2110048	51.93
Bin88	Actinobacteria;Propionibacteriales; Nocardioidaceae;Nocardioides	58	502	485	46.35	432204	8.17
Bin89	Bacteroidetes; Flavobacteria; Flavobacteriales	119	1626	1590	41.10	1636314	11.46
Bin94	Gammaproteobacteria; Oceanospirillales; SUP05 cluster	46	1297	1255	38.89	1156316	71.87
Bin95-1	Gammaproteobacteria; Alteromonadales;Alteromonadaceae	106	1342	1295	36.93	1151967	15.00
Bin95-2	Gammaproteobacteria; Alteromonadales;Psychromonadaceae	61	1592	1545	36.65	1473436	37.73
Bin98-1	Deferribacteres;Deferribacteres;Deferribacterales;SAR406 clade	129	2067	2017	32.81	1910366	12.58
Bin98-2	Deferribacteres;Deferribacteres;Deferribacterales;SAR406 clade	19	1014	973	33.28	946895	64.27
Bin100	Gammaproteobacteria;Oceanospirillales;OM182 clade	42	4111	3991	49.38	4327955	17.11

Bin102	Planctomycetes; Planctomycetia; Planctomycetales; Planctomycetaceae; Blastopirellula	280	7306	7067	48.28	8948294	19.31
Bin105-1	Bacteroidetes; Cytophagia; Cytophagales; Flammeovirgaceae; Marinoscillum	77	1994	1930	30.00	1803301	19.74
Bin105-2	Bacteroidetes; Cytophagia; Cytophagales; Flammeovirgaceae; Marinoscillum	62	1091	1064	29.29	930543	9.47
Bin108-2	Bacteroidetes; Flavobacteria; Flavobacteriales;F lavobacteriaceae	39	342	334	28.87	290756	53.88
Bin109	Gammaproteobacteria	158	1982	1943	53.48	1832546	11.60
Bin110-1	Gammaproteobacteria	277	7684	7335	49.57	7316535	14.56
Bin110-2	Gammaproteobacteria	319	6080	5741	51.53	5407492	65.81

Table S3. Summary of genome reconstruction completeness, contamination, and strain heterogeneity based on CheckM package. For detailed information of individual genomic bins see table S2.

Bin	Taxonomy	Markers	Marker sets	0	1	2	3	4	5+	Completeness	Contamination	Strain heterogeneity
Bin37	Alphaproteobacteria; Rhodospirillales; Rhodospirillaceae; Rhodospirillum	150	108	5	116	29	0	0	0	96.23	15.43	48.28
Bin38	Gammaproteobacteria; Oceanospirillales; Alcanivoracaceae; Alcanivorax	104	58	75	28	1	0	0	0	30.8	1.72	0
Bin39	Planctomycetes; Planctomycetia; Planctomycetales; Planctomycetaceae; Planctomyces	145	90	24	120	1	0	0	0	79.1	1.11	0
Bin40	Verrucomicrobia; Verrucomicrobiae; Verrucomicrobiales; Verrucomicrobia subdivision 3	104	58	70	32	2	0	0	0	44.43	3.45	0
Bin41	Nitrospirae; Nitrospira; Nitrospirales; Nitrospiraceae; Leptospirillum	104	58	2	79	23	0	0	0	96.55	12.54	0
Bin42-1	Alphaproteobacteria; Sphingomonadales; Erythrobacteraceae; Erythrobacter	570	294	141	427	2	0	0	0	78.73	0.45	50
Bin42-2	Alphaproteobacteria; Sphingomonadales; Erythrobacteraceae; Erythrobacter	570	294	10	535	25	0	0	0	98.95	5.03	8
Bin43	Alphaproteobacteria;Rhodobacterales; Hyphomonadaceae;Henriciella	530	309	63	446	21	0	0	0	89.22	4.29	9.52
Bin44	Planctomycetes; Planctomycetia; Planctomycetales; Planctomycetaceae	144	89	5	138	0	1	0	0	95.44	2.25	0
Bin45	Gammaproteobacteria; Oceanospirillales; Alcanivoracaceae; Alcanivorax	449	270	2	446	1	0	0	0	99.51	0.37	0
Bin46	Bacteroidetes; Cytophagia; Rhodothermaceae; Rubricoccus	275	184	85	187	3	0	0	0	69.33	0.65	0
Bin47	Actinobacteria; Actinobacteridae; Actinomycetales;Micrococo	401	199	157	242	2	0	0	0	60.11	0.54	0

	ccineae; Microbacteriaceae; Microbacterium											
Bin48	Verrucomicrobia; Verrucomicrobiae; Verrucomicrobiales	104	58	37	9	56	2	0	0	56.63	15.56	75.81
Bin49	Verrucomicrobia; Verrucomicrobiae; Verrucomicrobiales;Verrucomicrobiaceae; Verrucomicrobium	231	149	79	150	2	0	0	0	71.06	0.5	0
Bin50	Actinobacteria; Actinobacteria; Acidimicrobiales; Acidimicrobiaceae; Acidimicrobium	171	117	4	165	2	0	0	0	96.58	1.28	0
Bin51	Actinobacteria; Actinobacteria; Micrococcales; Microbacteriaceae; Microbacterium	148	92	103	43	2	0	0	0	22.05	2.17	0
Bin52-1	Deltaproteobacteria	248	156	22	224	2	0	0	0	88.41	0.68	50
Bin52-2	Deltaproteobacteria	248	156	11	237	0	0	0	0	93.91	0	0
Bin53	Planctomycetes; Planctomycetia; Planctomycetales; Planctomycetaceae	144	89	1	131	12	0	0	0	98.88	11.24	8.33
Bin54	Firmicutes; Clostridia; Clostridiales	148	92	87	59	2	0	0	0	32.48	1.15	0
Bin55	Proteobacteria;Deltaproteobacteria	248	156	12	231	5	0	0	0	93.38	3.21	0
Bin56	Proteobacteria;Deltaproteobacteria	248	156	12	230	6	0	0	0	93.7	2.5	0
Bin57	Deferribacteres; Deferribacteres; Deferribacterales	148	92	11	123	13	1	0	0	90.09	15.22	6.25
Bin58	Deltaproteobacteria; SAR324 clade	191	120	13	177	1	0	0	0	90.92	0.83	0
Bin59	Chloroflexi; SAR202 clade	161	108	8	152	1	0	0	0	92.59	0.93	100
Bin60	Alphaproteobacteria	349	230	52	288	8	1	0	0	83.07	2.1	0
Bin62	Planctomycetes; Planctomycetacia; Planctomycetales; Planctomycetaceae	150	108	8	3	79	55	5	0	92.59	10.28	4.01
Bin63	Actinobacteria; Actinobacteria; Actinomycetales	189	126	21	166	2	0	0	0	85.05	0.85	0
Bin64	Planctomycetes; OM190	150	108	10	104	36	0	0	0	91.2	19.6	0
Bin65	Gammaproteobacteria; Methylococcales; Methylococcaceae	104	58	76	28	0	0	0	0	39.66	0	0
Bin66	Gammaproteobacteria; Methylococcales; Methylococcaceae;	583	291	3	572	8	0	0	0	99.71	1.95	0

	Methylobacter											
Bin67-1	Bacteroidetes; Flavobacteria; Flavobacteriales; Flavobacteriaceae	279	187	20	248	11	0	0	0	90.91	4.99	0
Bin67-2	Bacteroidetes; Flavobacteria; Flavobacteriales; Flavobacteriaceae	279	187	40	227	12	0	0	0	88.45	4.9	50
Bin68	Gemmatimonadetes; Gemmatimonadetes; Gemmatimonadales;Gemmatimonadaceae	148	92	15	130	3	0	0	0	85.81	3.26	0
Bin70	Actinobacteria; Actinobacteria; Actinomycetales ;Mycobacteriaceae	104	58	103	1	0	0	0	0	1.72	0	0
Bin71	Chloroflexi; Thermomicrobia; Thermomicrobiales; Thermomicrobiaceae	161	108	57	102	2	0	0	0	56.74	1.85	0
Bin72	Chloroflexi; Dehalococcoidia; Dehalococcoidales;Dehalococcoidaceae; Dehalococcoides	150	108	34	20	94	1	1	0	70.83	8.26	20.39
Bin73	Gammaproteobacteria; Alteromonadales; Alteromonadaceae; SAR92 clade	452	271	26	424	2	0	0	0	92.81	0.74	100
Bin74-1	Gammaproteobacteria; Methylococcales; Methylococcaceae; Methylomicrobium	583	291	28	545	8	2	0	0	93.13	2.41	14.29
Bin74-2	Gammaproteobacteria; Methylococcales; Methylococcaceae; Methylomicrobium	583	291	67	501	15	0	0	0	85.49	2.25	0
Bin76	Gammaproteobacteria;Thiotrichales;Thiotrichaceae	546	285	35	466	45	0	0	0	96.25	8.01	0
Bin77	Betaproteobacteria; Methylophilales; Methylophilaceae; Methylotenera	104	58	98	6	0	0	0	0	8.62	0	0
Bin78	Gammaproteobacteria	276	175	46	218	12	0	0	0	79.86	5.43	8.33
Bin79	Gammaproteobacteria	276	175	51	193	28	4	0	0	77.1	15.29	2.5
Bin80	Gammaproteobacteria; Oceanospirillales; SUP05 cluster	102	56	15	51	36	0	0	0	91.72	26.3	25
Bin81	Planctomycetes; Pla3 lineage	144	89	7	136	1	0	0	0	92.13	0.07	100
Bin82-1	Gammaproteobacteria; Oceanospirillales; SUP05 cluster	102	56	2	53	17	18	12	0	98.21	28.9	5.59
Bin82-2	Gammaproteobacteria;	243	152	61	162	20	0	0	0	68.52	8.49	25

	Oceanospirillales; SUP05 cluster											
Bin84	Verrucomicrobia	230	148	5	184	38	2	1	0	99.8	26.35	0
Bin85	Alphaproteobacteria; Rhodospirillales; Rhodospirillaceae	337	201	20	292	25	0	0	0	91.87	6.9	4
Bin86	Nitrospirae;Nitrospira;Nitrospirales;Nitrospiraceae	104	58	10	38	51	5	0	0	86.21	19.83	3.03
Bin88	Actinobacteria;Propionibacteriales; Nocardioidaceae;Nocardioides	56	24	55	1	0	0	0	0	4.17	0	0
Bin89	Bacteroidetes; Flavobacteria; Flavobacteriales	104	58	7	80	15	2	0	0	88.79	6.66	4.76
Bin94	Gammaproteobacteria; Oceanospirillales; SUP05 cluster	56	24	45	1	1	4	0	5	9.38	20.83	9.35
Bin95-1	Gammaproteobacteria; Alteromonadales;Alteromonadaceae	102	58	11	44	45	2	0	0	81.9	19.98	3.92
Bin95-2	Gammaproteobacteria; Alteromonadales;Psychromonadaceae	276	175	34	241	1	0	0	0	84.43	0.14	0
Bin98-1	Deferribacteres;Deferribacteres;Deferribacterales;SAR406 clade	148	92	41	105	2	0	0	0	74.65	2.17	0
Bin98-2	Deferribacteres; Deferribacteres;Deferribacterales;SAR406 clade	148	92	7	141	0	0	0	0	92.39	0	0
Bin100	Gammaproteobacteria;Oceanospirillales;OM182 clade	150	108	68	82	0	0	0	0	51.01	0	0
Bin102	Planctomycetes; Planctomycetia; Planctomycetales; Planctomycetaceae; Blastopirellula	56	24	0	0	17	26	13	0	99	13.03	5.78
Bin105-1	Bacteroidetes; Cytophagia; Cytophagales; Flammeovirgaceae; Marinoscillum	148	92	13	122	13	0	0	0	90.22	11.48	7.69
Bin105-2	Bacteroidetes; Cytophagia; Cytophagales; Flammeovirgaceae; Marinoscillum	276	175	85	184	7	0	0	0	70.91	3.71	14.29
Bin108-2	Bacteroidetes; Flavobacteria; Flavobacteriales;Flavobacteriaceae	572	304	458	111	2	1	0	0	18.36	1.32	0
Bin109	Gammaproteobacteria	262	165	93	168	1	0	0	0	58.08	0.2	0
Bin110-1	Gammaproteobacteria	104	58	2	75	27	0	0	0	96.55	19.83	11.11
Bin110-2	Gammaproteobacteria	452	271	34	291	122	5	0	0	91.11	29.56	1.46

Table S4. The gene abundance of extracellular peptidases (EP) and carbohydrate metabolizing enzymes (auxiliary activities, AA; carbohydrate esterases, CE; glycoside hydrolases, GH; and polysaccharide lyases, PLs) in each bacteria genome and their transcripts in the GB plume and background.

		Gene abundance (#/Mb)					Transcripts in plume					Transcripts in background				
Taxonomy	# Bin	EP	AA	CE	GH	PL	EP	AA	CE	GH	PL	EP	AA	CE	GH	PL
Actinobacteria;Actinobacteria;Acidimicrobiales;Acidimicrobiaceae;Acidimicrobium	Bin50	3.2	1.1	1.1	3.7	0.0	1258.8	595.6	1471.2	2145.1	0.0	71.1	0.0	91.1	44.8	0.0
Actinobacteria;Actinobacteria;Actinomycetales	Bin63	0.0	0.7	1.3	4.0	0.0	0.0	361.2	3117.8	5750.9	0.0	0.0	235.9	640.7	2186.1	0.0
Actinobacteria;Actinobacteria;Actinomycetales;Mycobacteriaceae	Bin70	0.6	0.6	2.8	2.8	0.0	867.2	205.4	3147.1	966.7	0.0	517.7	208.0	2188.9	1507.4	0.0
Actinobacteria;Actinobacteria;Micrococcales;Microbacteriaceae;Microbacterium	Bin51	4.6	1.8	5.5	5.5	0.0	516.5	0.0	783.1	814.2	0.0	419.5	286.8	410.9	283.2	0.0
Actinobacteria;Actinobacteridae;Actinomycetales;Micrococcineae;Microbacteriaceae;Microbacterium	Bin47	0.6	1.9	0.0	3.1	0.0	0.0	0.0	0.0	0.0	0.0	0.0	0.0	0.0	0.0	0.0
Actinobacteria;Propionibacteriales;Nocardioidaceae;Nocardioides	Bin88	6.9	0.0	0.0	2.3	0.0	1095.8	0.0	0.0	113.6	0.0	72.5	0.0	0.0	49.3	0.0
Bacteroidetes;Cytophagia;Cytophagales;Flammeovirgaceae;Marinoscillum	Bin105-1	0.0	1.1	1.7	2.2	0.0	0.0	0.0	226.7	0.0	0.0	0.0	101.8	88.3	217.5	0.0
Bacteroidetes;Cytophagia;Cytophagales;Flammeovirgaceae;Marinoscillum	Bin105-2	0.0	1.1	1.1	2.1	0.0	0.0	0.0	0.0	0.0	0.0	0.0	0.0	0.0	0.0	0.0
Bacteroidetes;Cytophagia;Rhodothermaceae;Rubricoccus	Bin46	7.3	1.1	2.9	7.3	1.1	47.2	0.0	0.0	120.4	0.0	0.0	0.0	0.0	0.0	0.0
Bacteroidetes;Flavobacteria;Flavobacteriales	Bin89	2.4	0.0	1.8	9.2	0.0	84.8	0.0	0.0	1020.2	0.0	55.2	0.0	286.2	241.6	0.0
Bacteroidetes;Flavobacteria;Flavobacteriales;Flavobacteriaceae	Bin108-2	3.4	0.0	3.4	6.9	0.0	147.6	0.0	0.0	0.0	0.0	0.0	0.0	57.6	50.5	0.0
Bacteroidetes;Flavobacteria;Flavobacteriales;Flavobacteriaceae	Bin67-1	6.8	0.0	1.2	6.8	0.0	646.9	0.0	0.0	133.0	0.0	424.6	0.0	66.9	257.8	0.0
Bacteroidetes;Flavobacteria;Flavobacteriales;Flavobacteriaceae	Bin67-2	5.4	0.0	2.0	9.5	0.0	0.0	0.0	0.0	147.3	0.0	332.3	0.0	190.6	994.3	0.0
Chloroflexi;Dehalococcoidia;Dehalococcoidales;Dehalococcoidaceae;Dehalococcoides	Bin72	1.2	0.4	1.2	4.3	0.0	817.3	115.9	203.5	5176.8	0.0	759.2	75.5	241.3	1724.7	0.0
Chloroflexi;SAR202 clade	Bin59	0.0	0.3	4.3	1.0	0.0	0.0	2883.1	17489.3	4776.4	0.0	0.0	740.8	5952.0	2431.6	0.0
Chloroflexi;Thermomicrobia;Thermomicrobiales;Thermomicrobiaceae	Bin71	0.9	1.7	1.7	4.3	0.0	324.0	795.7	1788.6	2089.8	0.0	70.3	122.0	212.4	383.0	0.0

Deferribacteres;Deferribacteres;Deferribacterales	Bin57	0.5	0.2	2.0	10.2	0.7	611.7	253.4	2577.3	20273.8	397.0	514.1	137.5	1021.2	6559.5	514.6
Deferribacteres;Deferribacteres;Deferribacterales;SAR406clade	Bin98-1	0.5	0.5	1.0	5.2	0.0	0.0	0.0	0.0	0.0	0.0	0.0	520.6	87.5	198.5	0.0
Deferribacteres;Deferribacteres;Deferribacterales;SAR406clade	Bin98-2	4.2	0.0	3.2	0.0	0.0	235.1	0.0	0.0	0.0	0.0	421.5	0.0	89.2	0.0	0.0
Firmicutes;Clostridia;Clostridiales	Bin54	0.8	0.0	0.0	5.9	0.0	1134.9	0.0	0.0	1752.4	0.0	140.8	0.0	0.0	542.8	0.0
Gemmatimonadetes; Gemmatimonadetes; Gemmatimonadales; Gemmatimonadaceae	Bin68	9.2	0.4	2.8	6.0	0.0	10710.5	279.0	2556.7	5612.5	0.0	5932.7	48.5	1383.9	2876.1	0.0
Nitrospirae;Nitrospira;Nitrospirales;Nitrospiraceae	Bin86	0.5	0.0	0.9	3.8	0.0	671.9	0.0	853.1	6965.0	0.0	116.7	0.0	241.6	1001.2	0.0
Nitrospirae;Nitrospira;Nitrospirales;Nitrospiraceae;Leptospirillum	Bin41	2.2	0.0	0.9	3.1	0.0	5599.2	0.0	8988.4	6380.6	0.0	0.0	0.0	43.7	0.0	0.0
Planctomycetes;OM190	Bin64	4.5	0.3	4.0	8.1	0.0	6496.5	2829.1	3798.6	7836.4	0.0	1869.4	409.4	1159.2	2243.9	0.0
Planctomycetes;Pla3lineage	Bin81	2.7	0.7	1.7	3.7	0.0	14357.9	9771.6	7175.0	27155.0	0.0	1638.8	515.6	333.4	2838.7	0.0
Planctomycetes;Planctomycetacia;Planctomycetales;Planctomycetaceae	Bin62	2.4	0.2	4.2	13.4	0.2	5396.3	711.8	4182.1	15606.9	419.9	1147.2	61.8	1567.7	3656.5	0.0
Planctomycetes;Planctomycetia;Planctomycetales;Planctomycetaceae	Bin44	4.9	0.0	3.8	6.7	0.0	7053.1	0.0	9164.6	8516.8	0.0	1095.1	0.0	964.1	2061.9	0.0
Planctomycetes;Planctomycetia;Planctomycetales;Planctomycetaceae	Bin53	3.8	0.0	1.4	4.4	0.0	5223.6	0.0	1467.3	7368.6	0.0	1081.8	0.0	174.3	1472.1	0.0
Planctomycetes;Planctomycetia;Planctomycetales;Planctomycetaceae;Blastopirellula	Bin102	2.8	0.0	3.7	12.3	0.2	9570.7	0.0	10379.3	39990.1	865.5	988.2	0.0	1060.3	3709.5	0.0
Planctomycetes;Planctomycetia;Planctomycetales;Planctomycetaceae;Planctomyces	Bin39	3.0	0.3	4.4	12.0	0.0	5797.5	1396.8	3826.5	13444.1	0.0	453.6	0.0	116.1	976.5	0.0
Proteobacteria;Alphaproteobacteria	Bin60	4.1	0.4	2.9	3.3	0.4	0.0	0.0	0.0	0.0	0.0	0.0	0.0	0.0	0.0	0.0
Proteobacteria;Alphaproteobacteria;Rhodobacterales;Hyphomonadaceae;Henriciella	Bin43	0.3	0.3	0.7	0.3	0.0	0.0	0.0	0.0	0.0	0.0	0.0	133.3	0.0	0.0	0.0
Proteobacteria;Alphaproteobacteria;Rhodospirillales;Rhodospirillaceae	Bin85	1.0	2.4	1.7	2.8	0.0	133.7	1010.2	101.1	1297.0	0.0	58.1	483.8	228.8	433.0	0.0
Proteobacteria;Alphaproteobacteria;Rhodospirillales;Rhodospirillaceae;Rhodospirillum	Bin37	4.7	0.0	7.8	0.0	0.0	567.5	0.0	878.2	0.0	0.0	59.5	0.0	84.8	0.0	0.0
Proteobacteria;Alphaproteobacteria;Sphingomonadales;Erythrobacteraceae;Erythrobacter	Bin42-1	8.3	1.2	1.2	5.1	0.4	442.9	0.0	0.0	733.4	0.0	3177.0	499.7	0.0	1452.2	0.0
Proteobacteria;Alphaproteobacteria;Sphingomonadales;Erythro	Bin42-2	9.1	0.9	1.9	4.7	0.3	419.1	59.4	114.7	161.9	0.0	19.0	0.0	0.0	120.7	0.0

bacteraceae;Erythrobacter																
Proteobacteria;Betaproteobacteria;Methylophilales;Methylophilaceae;Methylotenera	Bin77	2.9	0.0	0.0	0.0	0.0	1901.5	0.0	0.0	0.0	0.0	190.5	0.0	0.0	0.0	0.0
Proteobacteria;Deltaproteobacteria	Bin52-1	1.2	0.7	0.7	5.7	0.0	952.3	2985.5	569.4	3928.3	0.0	234.9	882.0	305.2	1233.2	0.0
Proteobacteria;Deltaproteobacteria	Bin52-2	1.3	0.8	0.3	5.7	0.0	1455.0	263.6	227.8	4560.5	0.0	848.8	523.4	247.3	4345.8	0.0
Proteobacteria;Deltaproteobacteria	Bin55	0.6	0.2	2.1	6.2	0.0	698.6	0.0	398.0	4183.7	0.0	457.4	48.2	529.3	3662.4	0.0
Proteobacteria;Deltaproteobacteria	Bin56	1.0	0.8	1.8	5.4	0.0	1564.5	116.8	1967.1	3505.8	0.0	461.6	183.8	222.7	1787.3	0.0
Proteobacteria;Deltaproteobacteria;SAR324clade	Bin58	0.6	0.9	1.8	1.5	0.0	766.1	1537.2	2112.7	4968.5	0.0	360.9	389.0	1042.2	1962.5	0.0
Proteobacteria;Gammaproteobacteria	Bin109	1.1	1.6	1.6	1.6	0.0	722.4	398.1	2380.7	443.8	0.0	62.7	0.0	361.3	201.9	0.0
Proteobacteria;Gammaproteobacteria	Bin110-1	2.5	0.0	0.0	6.8	0.0	4030.3	0.0	0.0	16246.5	0.0	1045.0	0.0	0.0	3957.5	0.0
Proteobacteria;Gammaproteobacteria	Bin110-2	1.8	0.0	0.0	4.8	0.0	13214.8	0.0	0.0	15981.4	0.0	2978.9	0.0	0.0	4318.3	0.0
Proteobacteria;Gammaproteobacteria	Bin78	4.4	0.0	1.5	2.9	0.0	443.9	0.0	618.4	1036.5	0.0	3453.8	0.0	2103.0	640.2	0.0
Proteobacteria;Gammaproteobacteria	Bin79	3.2	0.0	1.6	3.2	0.0	833.4	0.0	1020.6	68.5	0.0	1117.4	0.0	1065.6	298.0	0.0
Proteobacteria;Gammaproteobacteria	Bin83	0.4	1.3	2.2	2.7	0.0	645.7	307.9	971.1	781.9	0.0	62.3	552.1	822.2	718.7	0.0
Proteobacteria;Gammaproteobacteria;Alteromonadales;Alteromonadaceae	Bin95-1	5.2	0.0	0.9	2.6	0.0	66.9	0.0	0.0	0.0	0.0	1879.3	0.0	314.0	36.1	0.0
Proteobacteria;Gammaproteobacteria;Alteromonadales;Alteromonadaceae;SAR92clade	Bin73	3.5	1.7	1.7	5.2	0.0	719.9	183.0	460.8	1131.6	0.0	127.8	100.7	0.0	252.8	0.0
Proteobacteria;Gammaproteobacteria;Alteromonadales;Psychromonadaceae	Bin95-2	3.4	0.0	0.0	3.4	0.0	431.6	0.0	0.0	321.6	0.0	514.8	0.0	0.0	581.4	0.0
Proteobacteria;Gammaproteobacteria;Methylococcales;Methylococcaceae	Bin65	1.4	0.0	0.0	4.2	0.0	7589.0	0.0	0.0	41434.4	0.0	2458.0	0.0	0.0	3726.7	0.0
Proteobacteria;Gammaproteobacteria;Methylococcales;Methylococcaceae;Methylobacter	Bin66	1.8	0.3	0.9	1.5	0.0	11711.5	5971.9	6154.1	6092.8	0.0	1599.3	270.5	0.0	151.9	0.0
Proteobacteria;Gammaproteobacteria;Methylococcales;Methylococcaceae;Methylomicrobium	Bin74-1	2.2	0.4	0.4	1.3	0.0	10168.2	467.5	1763.6	2876.9	0.0	4529.5	669.8	585.5	1616.7	0.0
Proteobacteria;Gammaproteobacteria;Methylococcales;Methylococcaceae;Methylomicrobium	Bin74-2	2.1	0.7	0.7	1.4	0.0	36848.7	1106.3	18881.4	6418.8	0.0	11086.6	672.4	5134.6	13305.0	0.0
Proteobacteria;Gammaproteobacteria;Oceanospirillales;Alcanivoracaceae;Alcanivorax	Bin38	3.5	3.0	0.5	4.5	0.0	607.2	316.1	111.9	543.8	0.0	0.0	0.0	0.0	0.0	0.0
Proteobacteria;Gammaproteobacteria;Oceanospirillales;Alcaniv	Bin45	3.1	1.7	1.4	2.8	0.3	1152.6	1369.0	2480.0	766.1	0.0	2475.5	1100.3	1876.6	719.9	0.0

oracaceae;Alcanivorax																
Proteobacteria;Gammaproteobacteria;Oceanospirillales;OM182clade	Bin100	4.9	1.4	2.1	6.7	0.0	4358.8	670.3	1935.2	5473.8	0.0	1235.5	175.1	793.7	1016.5	0.0
Proteobacteria;Gammaproteobacteria;Oceanospirillales;SUP05cluster	Bin80	0.6	2.5	1.8	2.5	0.0	0.0	464.2	0.0	242.4	0.0	0.0	0.0	0.0	0.0	0.0
Proteobacteria;Gammaproteobacteria;Oceanospirillales;SUP05cluster	Bin82-1	0.0	0.0	1.9	4.5	0.0	0.0	0.0	0.0	315.8	0.0	0.0	0.0	383.8	771.4	0.0
Proteobacteria;Gammaproteobacteria;Oceanospirillales;SUP05cluster	Bin82-2	0.0	0.0	1.2	6.0	0.0	0.0	0.0	5066.7	20686.2	0.0	0.0	0.0	269.4	5084.0	0.0
Proteobacteria;Gammaproteobacteria;Oceanospirillales;SUP05cluster	Bin94	0.0	3.5	3.5	3.5	0.0	0.0	1825.5	310.2	1572.3	0.0	0.0	280.4	82.0	23.3	0.0
Proteobacteria;Gammaproteobacteria;Thiotrichales;Thiotrichaceae	Bin76	3.7	0.4	0.4	1.6	0.0	1348.5	231.9	0.0	389.0	0.0	877.7	151.0	0.0	152.8	0.0
Verrucomicrobia	Bin84	0.8	0.0	2.2	13.0	0.8	319.1	0.0	527.8	4792.5	97.9	173.1	0.0	150.0	1571.8	0.0
Verrucomicrobia;Verrucomicrobiae;Verrucomicrobiales	Bin48	0.9	0.0	2.0	9.5	0.0	2430.2	0.0	4907.5	12720.2	0.0	576.9	0.0	2591.7	6288.8	0.0
Verrucomicrobia;Verrucomicrobiae;Verrucomicrobiales;Verrucomicrobiaceae;Verrucomicrobium	Bin49	0.0	0.0	2.0	12.7	0.0	0.0	0.0	1177.6	4783.2	0.0	0.0	0.0	583.9	3025.1	0.0
Verrucomicrobia;Verrucomicrobiae;Verrucomicrobiales;Verrucomicrobiasubdivision3	Bin40	0.6	0.3	3.1	10.8	0.3	0.0	209.4	1743.6	6388.8	261.2	82.2	22.7	557.5	1338.8	151.2

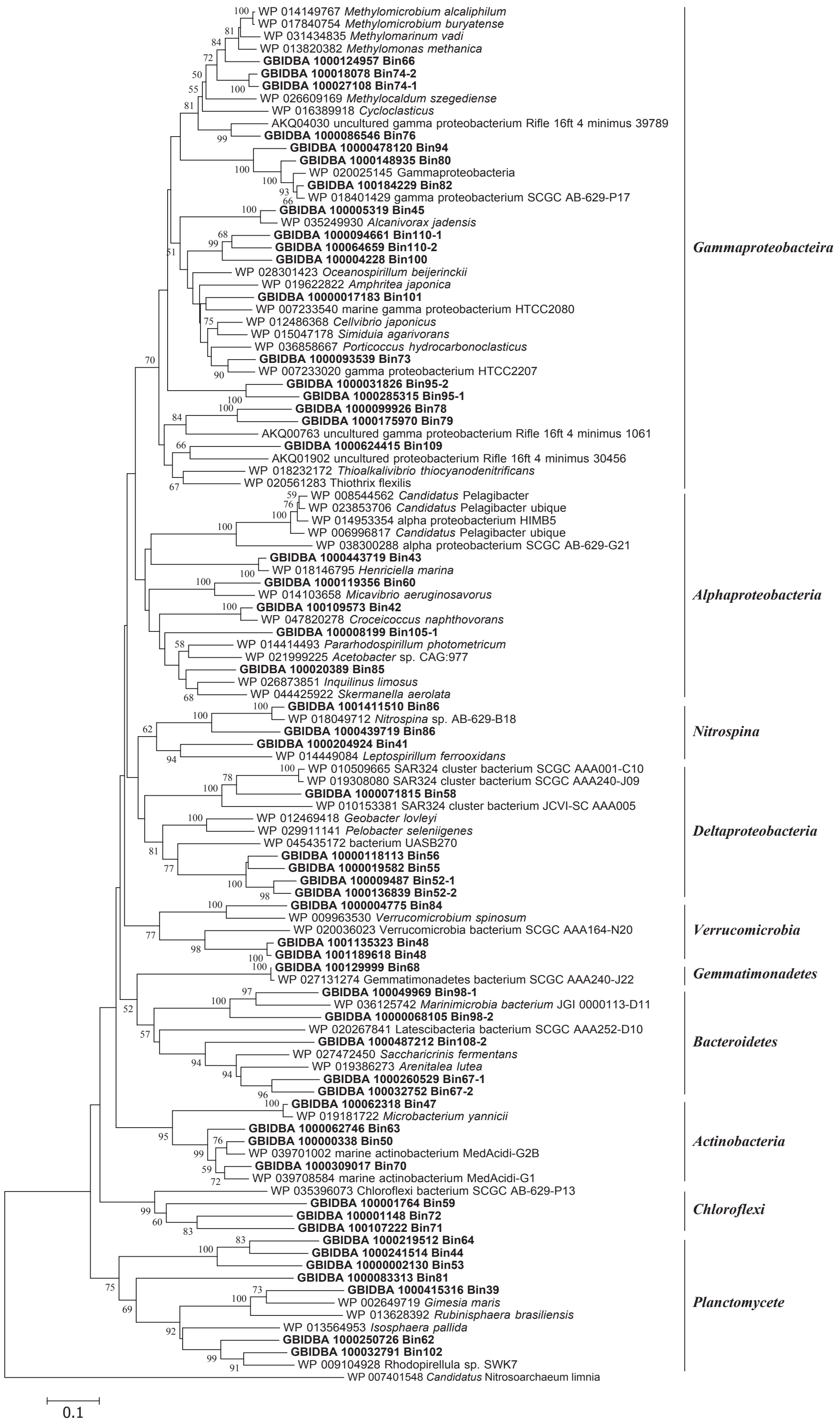

Figure S1. Phylogenetic tree of ribosomal protein S3 genes from bacterial genomic bins and their close related sequences in public database. This tree was generated using the maximum likelihood method (RAxML) with 1000 time bootstraps; only bootstraps >50% are shown in the tree.

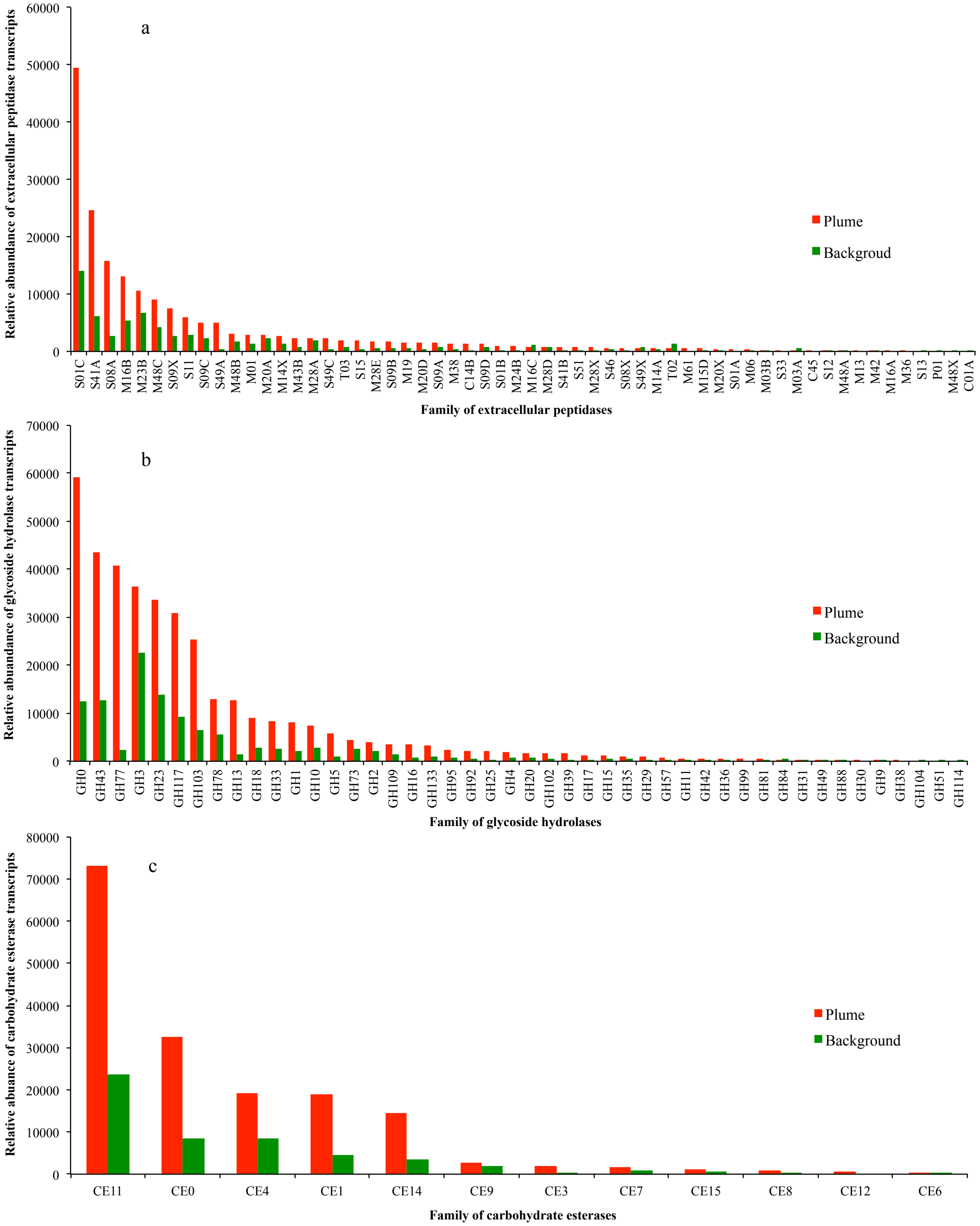
a
Relative abuandance of extracellular peptidase transcripts
60000
50000
40000
30000
20000
10000
0
Plume
Backgroud
S01C S41A S08A M16B M23B M48C S09X S11 S09C S49A M48B M01 M20A M14X M43B M28A S49C T03 S15 M28E S09B M19 M20D S09A M38 C14B S09D S01B M24B M16C M28D S41B S51 M28X S46 S08X S49X M14A T02 M61 M15D M20X S01A M06 M03B S33 M03A C45 S12 M48A M13 M42 M16A M36 S13 P01 M48X C01A
Family of extracellular peptidases
b
Relative abuandance of glycoside hydrolase transcripts
70000
60000
50000
40000
30000
20000
10000
0
Plume
Background
GH0 GH43 GH77 GH3 GH23 GH117 GH103 GH78 GH13 GH18 GH33 GH1 GH10 GH5 GH73 GH2 GH109 GH16 GH133 GH95 GH92 GH25 GH4 GH20 GH102 GH39 GH17 GH15 GH35 GH29 GH57 GH11 GH42 GH36 GH99 GH81 GH84 GH31 GH49 GH88 GH30 GH9 GH38 GH104 GH51 GH114
Family of glycoside hydrolases
c
Relative abuance of carbohydrate esterase transcripts
80000
70000
60000
50000
40000
30000
20000
10000
0
Plume
Background
CE11 CE0 CE4 CE1 CE14 CE9 CE3 CE7 CE15 CE8 CE12 CE6
Family of carbohydrate esterases

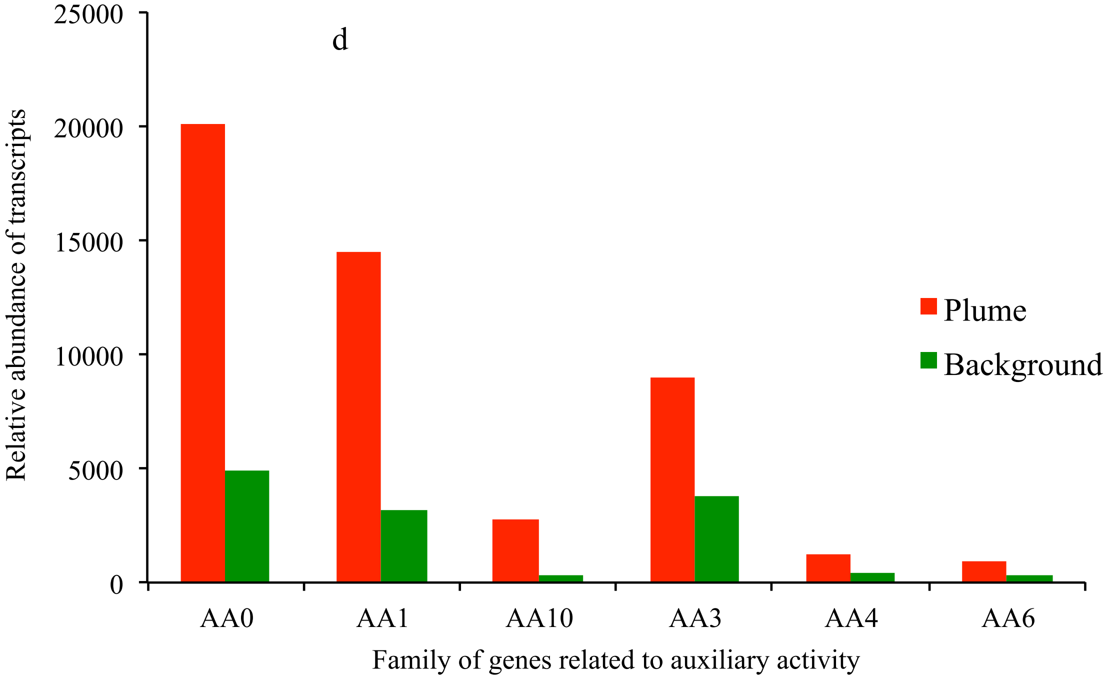

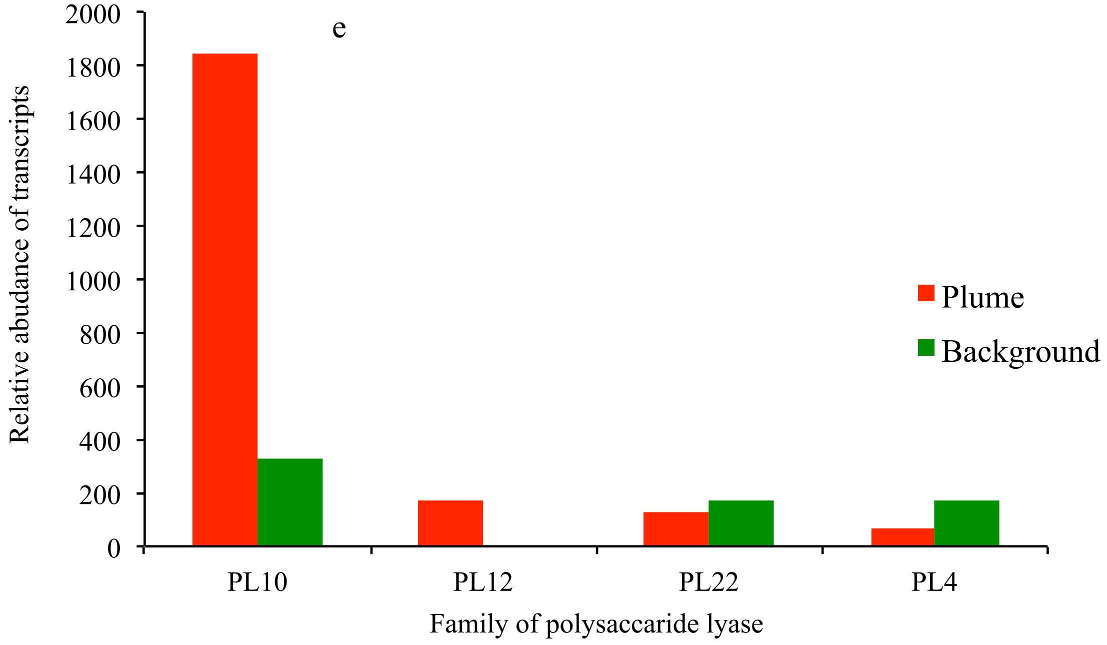

Figure S2. Relative abundance of transcripts at the level of families of genes encoding to extracellular peptidases (a), glycoside hydrolases (b), carbohydrate esterases (c), auxiliary activity enzymes (d) and polysaccharide lyases (e) identified in the GB plume (red bar) and in the background (green bar).

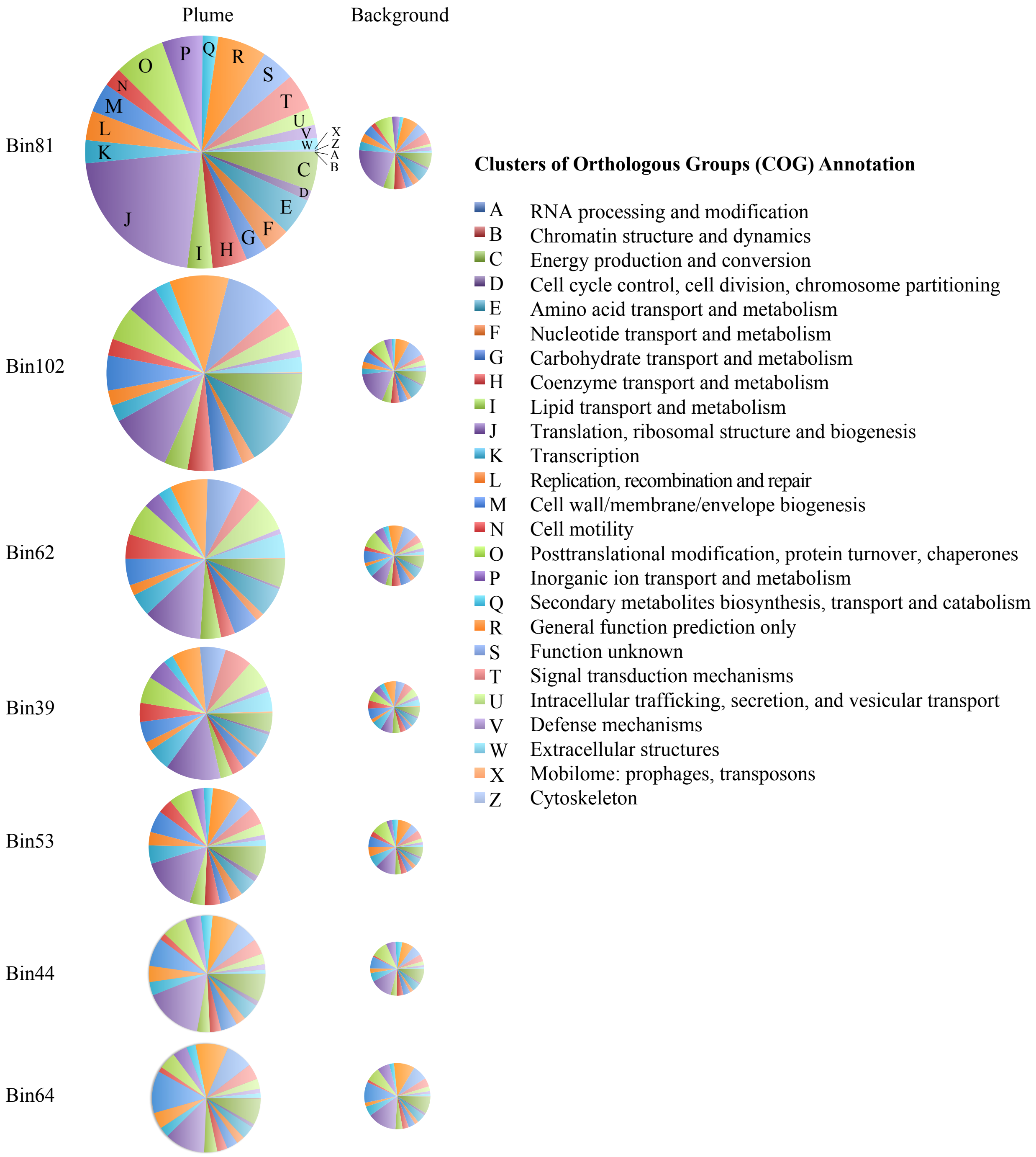

Figure S3. The transcript composition of seven members of Planctomycetes based on the functional prediction of COG in the GB plume and background. The size of the pie indicates the relative transcript abundance for each member of Planctomycetes.

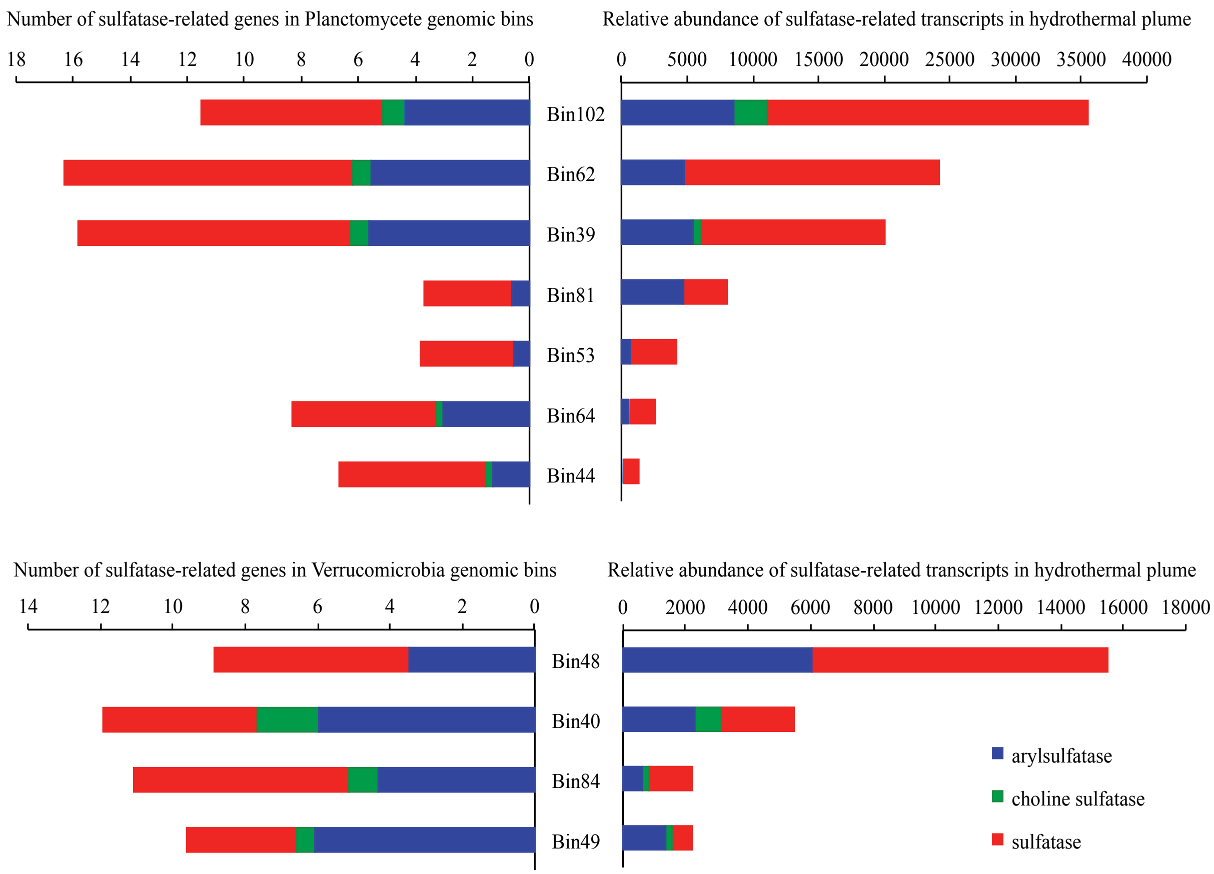

Figure S4. Relative abundance of sulfatase-related genes and their transcripts in deep-sea Planctomycetes (above panel) and Verrucomicrobia (bottom panel).
